# Insights into the Differential Preservation of Bone Proteomes in Inhumed and Entombed Cadavers from Italian Forensic Caseworks

**DOI:** 10.1021/acs.jproteome.1c00904

**Published:** 2022-03-22

**Authors:** Andrea Bonicelli, Aldo Di Nunzio, Ciro Di Nunzio, Noemi Procopio

**Affiliations:** † Forensic Science Research Group, Faculty of Health and Life Sciences, Applied Sciences, Northumbria University, NE1 8ST Newcastle Upon Tyne, United Kingdom; ‡ Chemical Sciences Department, University of Naples Federico II, 80126 Naples, Italy; § Legal Medicine Department, University of Catanzaro Magna Graecia, 88100 Germaneto, Italy

**Keywords:** bone, burial environment, post-mortem interval, age-at-death, forensics, post-translational protein modifications

## Abstract

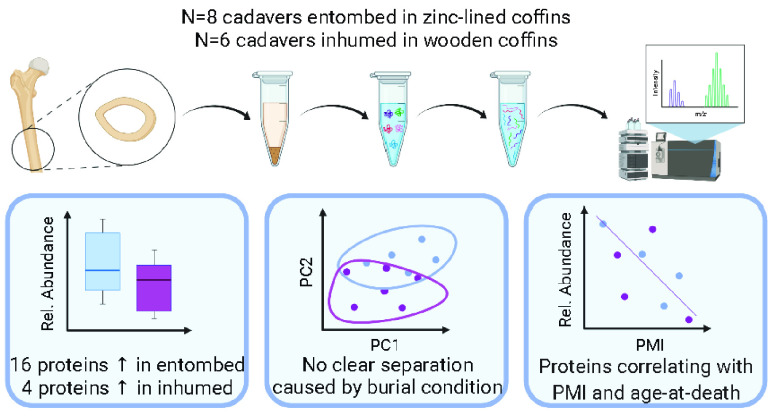

Bone is a hard biological tissue and a precious reservoir of information in forensic investigations as it retains key biomolecules commonly used for identification purposes. Bone proteins have recently attracted significant interest for their potential in estimating post-mortem interval (PMI) and age at death (AAD). However, the preservation of such proteins is highly dependent on intrinsic and extrinsic factors that can hinder the potential application of molecular techniques to forensic sciences. The present study aims at investigating the effects that two commonly used types of burial practices (entombment and inhumation) have on bone protein survival. The sample consists of 14 exhumed individuals from cemeteries in Southern Italy with different AADs (29–85 years) and PMIs (1–37 years). LC-MS/MS analyses show that 16 proteins are better preserved under the entombed conditions and 4 proteins are better preserved under the inhumed conditions, whereas no clear differences are detected for post-translational protein modifications. Furthermore, several potential “stable” protein markers (*i.e*., proteins not affected by the burial environment) are identified for PMI and AAD estimation. Overall, these results show that the two burial environments play a role in the differential preservation of noncollagenous proteins, confirming the potential of LC-MS/MS-based proteomics in forensic sciences.

## Introduction

The estimation of the post-mortem interval (PMI) represents one of the most complex tasks that forensic scientists are asked to perform during the examination of human remains. The complexity of the estimation relies on the fact that several factors can differentially affect the cadaveric decomposition, and for this reason, there are not universally accepted methods in the literature. Taphonomic changes are highly dependent on different environmental conditions (*e.g.*, temperature, precipitation, humidity, soil composition).^1,2^ Among those, the burial modality (*e.g.*, exposed, buried in the ground or in coffins, submerged in water) and the accessibility of the carcass to bacteria, insects, and scavengers play central roles in decomposition and its rate.^1,3,4^ There are also intrinsic biological factors that can affect post-mortem changes such as body size, age, and trauma.^5^ All of these variables must be considered when developing estimation methods for forensic purposes. One of the most commonly utilized methods for PMI estimation is the evaluation of the formation of adipocere.^6^ This method, however, becomes unreliable for extended PMIs or for fully skeletonized remains.^6^ Furthermore, environmental conditions (e.g., pH, humidity, temperature) and intrinsic factors (e.g., body fat) can heavily affect both the adipocere formation and the rate of decomposition.^7^ A recent study on 408 bodies showed that the presence of a zinc coffin can considerably slow the decay process, maintaining the body in a biologically and chemically stable stage for decades.^8^ This has been also shown to influence adipocere formation, an; therefore, it represents a substantial problem in the application of this methodology for PMI estimation.^9^

More recently, new molecular and biophysical methods have opened new routes for the estimation of PMI from skeletal remains. Among these techniques, vibrational and fluorescence spectroscopy have seen a considerable growth in the literature^10−12^ due to their high reproducibility and versatility. Biophysical methods involve the use of dispersive X-ray mapping, computed tomography, and scanning electron microscopy with energy-dispersive X-ray analysis (SEM-EDX) and have shown promising results.^12,13^ Among the biomolecular methods, proteomics is attracting great interest in the forensic community, despite representing a relatively new field that needs further evaluations and validations before its application to forensic investigations.^5,14−18^ The analysis of the proteome offers a large pool of potential biomarkers with different physicochemical and biological properties that could allow the development of statistical models capable of accounting for intrinsic and extrinsic taphonomic variables, reducing biases in PMI estimation, and increasing its accuracy.

In the attempt to evaluate the effects that different burial types and environment have on decomposition, an experimental model compared pigs’ adipose tissue samples that were buried directly in the soil (control sample), placed in mock coffins (lined with both plastic and satin material or unlined) and then buried in soil, wrapped in polyethylene bags and buried in soil, or wrapped in polyester and cotton clothes and buried in soil over a 12 month period. The results showed differential formation of adipocere when analyzed by infrared spectroscopy, inductively coupled plasma mass spectrometry, and gas chromatography–mass spectrometry.^19^ In particular, no adipocere was formed in plastic bags due to the absence of diffusion properties and the consequent liquefaction of the tissues. Similarly, no adipocere was found in the samples wrapped in cotton clothing. In contrast, polyester clothing induced the formation of adipocere, as supported by the accumulation of fatty acids. The authors suggested that this type of material facilitates the exchange of cations, inducing the saponification process.^19^ Finally, the adipose tissue samples buried in the two different types of coffins showed similar results, with a general delay in the formation of adipocere in comparison with the control environment and a higher decomposition rate.^19^ Overall, these studies show the importance of investigating the effect of different burial conditions when considering decomposition rates.

To better understand the context of the current study, it is essential to summarize the events that lead to cadaveric decomposition. Immediately after death, autolytic processes caused by the breakdown of cellular membranes result in the release of hydrolytic enzymes that lead to the digestion of the surrounding tissues.^20^ The corpse’s environment is then rapidly converted from aerobic to fully anaerobic. This change establishes the ideal conditions for anoxic bacteria from the gut, commonly referred to as endogenous bacteria or the thanatomicrobiome, to travel into the body and to proliferate rapidly.^21^ This marks the beginning of the putrefactive stage, at ∼1 h post-mortem, and continues throughout the next 48 h.^22^ During this stage, bacteria transmigrate to the rest of the body via the blood vessels^23,24^ and reach the skeletal system within a day of death. Reductive catalysis promoted by endogenous bacteria results in the bloating of the corpse, caused by the accumulation of gases in the inner body cavities.^25^ When the accumulation of gases applies sufficient pressure on the soft tissues, the abdomen is ruptured, and internal tissues are exposed. This situation offers an ideal environment for the colonization of the body by insects and by exogenous microbial communities present in the surrounding environment.^26−28^ The decomposing body is a natural reservoir of nutrients that enhances the proliferation of exogenous communities, which start to replace the endogenous bacterial population.^29^ With the progression of the decomposition, specific bacterial species are attracted in a quite consistent way, regardless of the type of burial environment and condition (*e.g.*, soil type, buried vs exposed).^30^ For this reason, microbial succession has been studied by several authors as a means of estimating the PMI.^30−33^ Despite the fact that it is well known that bacteria are the main drivers for bone bioerosion and the consequent protein decay, the origin (*e.g.*, endogenous, or exogenous) of the microorganisms responsible for the degradation of biomolecules is not yet fully clarified. Despite the fact that proteomics has been useful for successfully discriminating microbially versus nonmicrobially driven decomposition in an animal model,^34^ the specific role that endogenous and exogenous microbes may have in bone biomolecules still has to be elucidated, especially considering the lack of specific studies on human cadavers.

The present study aims to investigate bone proteome changes in relation to two different types of burial conditions commonly found in Italian cemeteries, namely, the entombment and the inhumation of cadavers. The samples analyzed are divided into two cohorts, one being corpses buried in zinc-lined coffins and placed in mausoleums in cemeteries (entombed) and the other being bodies placed in wooden coffins and buried in the ground (inhumed). It is reasonable to think that the first condition would reduce the access of the body to exogenous (soil) bacteria, allowing for the evaluation of the specific effects that endogenous bacteria have on proteins, whereas the second condition would offer access of the cadaver to both endogenous and exogenous bacteria as well as to insects and small scavengers. Thus the proteome should be better preserved in the first scenario compared with the second scenario. Understanding the proteome preservation in different burial conditions is crucial to the development of future robust biomarkers that could be used for PMI estimation. Therefore, the secondary aim of this work is to evaluate the presence of noncollagenous proteins (NCPs) that are only minorly affected, or not affected at all, by the different burial environments and that could be useful for estimating the PMI as well as the age-at-death (AAD) of the cadavers.

## Material and Methods

### Sample Composition

Fourteen male individuals (mean age 66.36 ± 17.64) were included in this study (Table 1). Eight individuals were buried in zinc-lined coffins (entombed, mean age 68.62 ± 19.65) with a PMI ranging between 1 and 30 years (mean PMI 9.88 ± 10.83). The remaining six were buried in wooden coffins (inhumed, mean age 63.33 ± 16.74) and with a PMI between 1 and 37 years (mean PMI 9.67 ± 13.47). The bone samples belonging to the deceased individuals subjected to this analysis were originally acquired from cemeteries in Campania (Italy) by one of the authors (C. D. N.) at the request of the Judicial Authority and preserved at the forensic laboratory of the Legal Medicine Department, University of Catanzaro Magna Graecia. Within the Italian juridic framework, the “Provision relating to the processing of particular categories of data, pursuant to art. 21, paragraph 1 of Legislative Decree 10 August 2018, n. 101” (Annex I, point 5.3) of the Italian Guarantor for the protection of personal data regulates the use of biological material or samples derived from it through laboratory analysis that have been taken for judicial purposes from deceased subjects. According to this provision, obtaining the informed consent of the deceased to use the samples for research purposes is not possible and therefore is not required, and permission from the legal next-of-kin is also not required. On these bases, we were guaranteed the ethical approval for the study (Northumbria University Ethics Committee ref. no. 16528). The specimens were obtained with an oscillating SG 700 saw (Schreiber instrument, Germany), and each one consisted of a 4.0 cm slice of the diaphyseal transverse section of the right mid femur. With a milling cutter (Dremel 200, Bosch USA), the outermost and innermost organic layers were removed, and the bone was cut into more fragments. These were washed in 50 mL of distilled water on an orbital agitator for 20 min, then air-dried; consequently, they were washed in 50 mL of 96% ethanol (EtOH) on an orbital agitator for 20 min, then air-dried again. Finally, they were washed in 50 mL of diethyl ether on an orbital agitator for 60 min and air-dried. The samples were cooled at −30 °C and subsequently pulverized with a tungsten carbide ball in a steel mill at room temperature (two repetitions of 3 min at 30 Hz) (TissueLyser equipped with grinding jar sets, QIAGEN, Germany). During the pulverization of different individual remains, the mill was rinsed with 10% v/v bleach followed by H_2_O and 96% v/v EtOH.

**Table 1:** Bone Sample Replicates Obtained from Each Individual and Their Corresponding Burial Condition, PMI, and Age at Death

sample code	burial condition	PMI (years)	age (years)
NP21_01A/B	zinc-lined coffin	30	74
NP21_02A/B	zinc-lined coffin	1	81
NP21_03A/B	zinc-lined coffin	24	85
NP21_04A/B	zinc-lined coffin	7	83
NP21_05A/B	zinc-lined coffin	4	65
NP21_06A/B	zinc-lined coffin	5	29
NP21_07A/B	zinc-lined coffin	3	81
NP21_08A/B	zinc-lined coffin	5	51
NP21_09A/B	wooden coffin	13	79
NP21_10A/B	wooden coffin^a^	37	65
NP21_11A/B	wooden coffin	3	82
NP21_12A/B	wooden coffin	1	45
NP21_13A/B	wooden coffin	1	42
NP21_14A/B	wooden coffin	3	67

a In this specific case, the cadaver has been moved from the inhumed environment, after being wrapped in a linen sheet, into a marble niche.

### Fourier Transform Infrared Spectroscopy (FTIR)

Infrared spectroscopy was employed to study the matrix composition of the samples. Data were collected by means of an ALPHA T Platinum spectrometer (Bruker Optics, Germany) in attenuated total reflectance mode. The range of interest was 2000–400 cm^–1^ with 4 cm^–1^ resolution for a total of 64 scans. Approximately 3 mg of bone powder was analyzed; in between each analysis, the holder and crystal were cleaned with deionized water. Spectral analysis was performed with Spectrum software 10.2 (PerkinElmer, USA). Semiquantitative analysis was carried out according to Kontoupolos et al.^35^ by calculating the peak heights from the baseline. The baseline for each peak of interest (amide I, v2CO_3_^2–^, v3PO_4_^3–^, and v4PO_4_^3^) was manually corrected when the automatic mode failed to identify the trough. The ratios considered were the infrared splitting factor (IRSF), carbonate to phosphate (C/P), type-B carbonate substitution (BPI), and amide to phosphate (Am/P).

### Protein Extraction

Twenty-five mg of bone powder from each of the 14 individuals was processed in duplicate (“A” and “B” samples) for protein extraction according to Procopio and Buckley.^36^ Each sample was decalcified with 1 mL of 10% v/v formic acid (Fisher Scientific, U.K.) for 6 h at 4 °C. After the acid soluble fraction was removed, the acid-insoluble fraction was incubated at 4 °C for 18 h in 500 μL of 6 M guanidine hydrochloride/100 mM TRIS buffer (pH 7.4, Sigma-Aldrich, U.K.). The buffer was exchanged into 100 μL of 50 mM ammonium acetate (Scientific Laboratory Supplies, U.K.) with 10K molecular-weight cut off filters (Vivaspin 500 poly(ether sulfone), 10 kDa, Sartorius, Germany), and the sample was reduced with 4.2 μL of 5 mM dithiothreitol (DTT) (Fluorochem, U.K.) for 40 min at room temperature before alkylation in 16.8 μL of 15 mM iodoacetamide (Sigma-Aldrich, U.K.) for 45 min in the dark at room temperature. Samples were then quenched with another 4.2 μL of 5 mM DTT and digested with 0.4 μg of trypsin (Promega, U.K.) for 5 h at 37 °C and finally frozen. With the addition of 15 μL of 1% v/v trifluoroacetic acid (TFA) (Fluorochem, U.K.), digestion was stopped, and the samples were desalted, concentrated, and purified using OMIX C18 pipet tips (Agilent Technologies, U.S.A.) with 0.1% v/v TFA as the washing solution and 50% v/v acetonitrile (ACN) (Thermo Fisher Scientific, U.K.)/0.1% v/v TFA as a conditioning solution. Pipette tips were conditioned using two volumes of 100 μL of 0.1% v/v TFA and washed twice with 100 μL of 50% v/v ACN/0.1% v/v TFA. The sample was then siphoned into the tip at least 10 times to efficiently bind peptides to the absorbent membrane. Two consecutive washing steps with 100 μL of 0.1% v/v TFA were performed prior to the peptide’s elution in 100 μL of 50% v/v ACN/0.1% v/v TFA. Purified peptides were left drying in the fume cupboard at room temperature prior to their LC-MS/MS analysis.

### LC/MS-MS Analysis

Samples were resuspended in 5% v/v ACN/0.1% v/v TFA and were analyzed by LC-MS/MS using an Ultimate 3000 Rapid Separation LC (RSLC) nano LC system (Thermo Corporation, Sunnyvale, CA) coupled to an Exploris 480 Quadrupole-Orbitrap Mass Spectrometer (Thermo Fisher Scientific, Waltham, MA). Peptides were separated on an EASY-Spray reverse-phase LC column (500 mm × 75 μm diameter (i.d.), 2 μm, Thermo Fisher Scientific) using a gradient from 96% v/v A (0.1% v/v FA in 3% v/v DMSO) and 2% v/v B (0.1% v/v FA in 80% v/v ACN 3% v/v DMSO) to 8, 30, and 50% v/v B at 14, 50, and 60 min, respectively, at a flow rate of 250 nL min^–1^. An acclaim PepMap 100 C18 LC column (5 mm × 0.3 mm i.d., 5 μm, 100 Å, Thermo Fisher Scientific) was used as a trap column at a flow rate of 10 μL min^–1^ maintained at 45 °C. The LC separation was followed by a cleaning cycle with an additional 15 min of column equilibration time. Then, peptide ions were analyzed in full-scan MS scanning mode at 60 000 MS resolution with an automatic gain control (AGC) of 3e6, injection time of 200 ms, and scan range of 375–1400 *m*/*z*. The top 20 most abundant ions were selected for data-dependent MS/MS analysis with a normalized collision energy (NCE) level of 30 performed at 17 500 MS resolution with an AGC of 1e5 and a maximum injection time of 100 ms. The isolation window was set to 2.0 *m*/*z*, with an underfilled ratio of 0.4%, and dynamic exclusion was employed; thus one repeat scan (i.e., two MS/MS scans in total) was acquired in a 25 s repeat duration, with the precursor being excluded for the subsequent 25 s.

### Data and Statistical Analysis

The group differences between FTIR parameters were evaluated by means of the *t*-test.

Peptide mass spectra were searched against the SwissProt_2021_03 database (selected for *Homo sapiens*, 20 386 entries) using the Mascot search engine (version 2.8.0; www.matrixscience.com) for matches to primary protein sequences. This search included the fixed carbamidomethyl modification of cysteine (+57.02 Da), as it is induced from the addition of DTT to the proteins. The deamidation of asparagine and glutamine (+0.98 Da) was considered to be a variable modification together with oxidation (methionine, proline, and lysine, +15.99 Da) due to the interest in their relationship with the post-mortem processes. The enzyme was set to trypsin with a maximum of two missed cleavages allowed. Mass tolerances for precursor and fragmented ions were set at 5 and 10 ppm, respectively. It was assumed that all spectra held either 2+ or 3+ charged precursors. Progenesis Qi for Proteomics (version 4.1; Nonlinear Dynamics, Newcastle, U.K.) was used to perform relative quantitation calculations using the recorded ion intensities (area under the curve (AUC)) and averaging the *N* most abundant peptides for each protein (Hi-N method, where *N* = 3) and PTM identifications. To increase the reliability of the matches, we excluded peptide ions with a score of <29, which indicates identity or extensive homology (false discovery rate (FDR) at *p* < 0.05), from the analysis based on the Mascot evaluation of the peptide score distribution for the searched .mgf file originating from Progenesis (combining all of the samples in a single experiment). Proteins with a unique peptide count of <2 were excluded from subsequent analyses. Furthermore, protein abundances were normalized using log2 transformation, and robust empirical Bayes regression (ComBat)^37^ was applied to normalize the batch effects between A and B biological replicates. Data analysis on normalized parameters accounted for principal component analysis (PCA) to evaluate differences between the different burial sites for all proteins quantified by LC-MS/MS and those for selected deamidated peptides that provided abundances different from zero in all samples. The differences between burial conditions for each protein were further evaluated by means of the *t*-test. Plots were created using R studio version 1.3.959. STRING software version 11.0 was used to visualize functional links between the extracted proteins.^38^ The relationship between proteins, age at death, and PMI was evaluated by Spearman’s rank correlation coefficient with significance set at *p* < 0.05 due to the monotonic but not linear association between variables.

The post-translational modification (PTM) analysis focused on peptide deamidation, as this was previously found to be correlated with both age and PMI.^4,39^ Only peptides presenting both a nondeamidated and a deamidated form and with an abundance different from zero for each sample were used in the quantification of the modification percentage according to the eq 1.^3^ The same statistical exploration employed for proteins was also applied to evaluate the PTMs among the different burial conditions, ages, and PMIs.
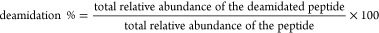
1

## Results

**Figure 1:**
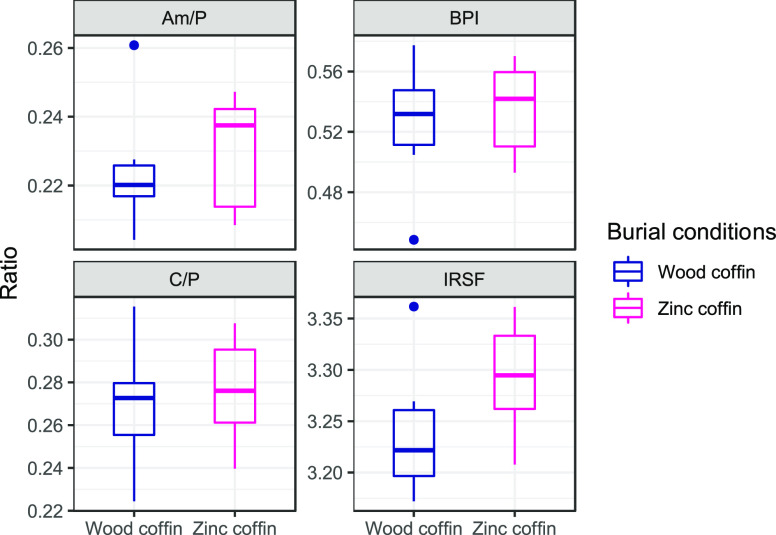
Boxplot showing the four parameters evaluated with FTIR. Higher values are observed for the wooden coffin group (in blue) compared with the zinc-lined coffin group (in magenta) (reported, respectively, as “wood coffin” and “zinc coffin” in the figure).

FTIR analyses of bone matrix composition do not show any significant difference between the two burial conditions. However, the mean values for C/P (*R* = 0.66) and IRSF (*R* = 0.58) are visibly higher in the individuals buried in zinc-lined coffins. The same trend is true for Am/P (*R* = 0.58) and BPI (*R* = 0.61), despite the fact that the qualitative differences in this case are smaller than those observed for the previous two parameters (Figure 1).

### Effect of Burial Condition of Protein Survival

The LC-MS/MS analysis allowed for the acquisition of 92 619 spectra that resulted in the matching of 6328 ions and the final retainment, after all of the filtration steps previously explained, of 90 proteins.

PCA was employed to understand whether there were differences in the protein relative abundances between the inhumed and the entombed conditions.

**Figure 2:**
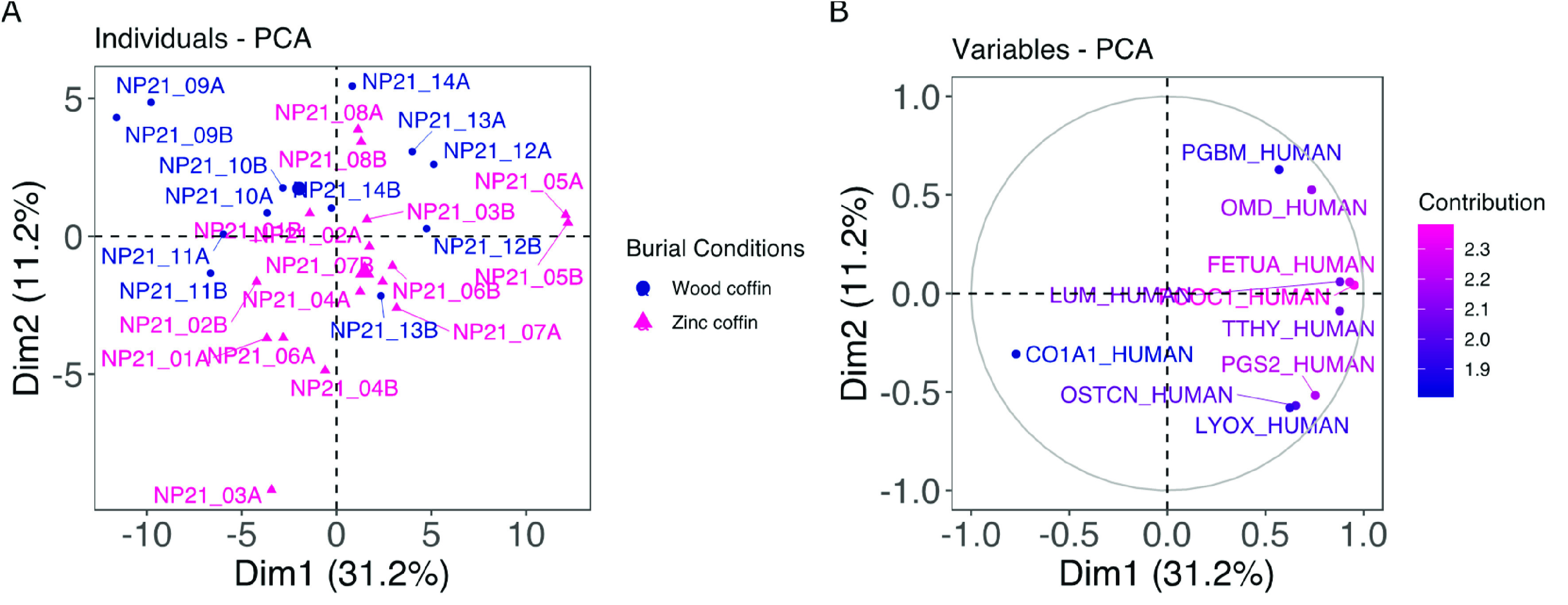
PCA plot showing (A) the individual clustering according to the two burial conditions (blue dots for wooden coffins and magenta triangles for zinc-lined coffins, reported, respectively, as “wood coffin” and “zinc coffin” in the figure) and (B) the relative abundance of the 10 proteins that provided the highest contribution in the clustering.

There are not clear clusters separating the two groups and the cumulative variance does not reach 50%, being ∼42% for the first two dimensions. However, a separation among the groups is visible on the second component, with the exclusion of the individual NP21_08, a 51-year-old male with a PMI of 5 years buried in a zinc-lined coffin. Most of the variance (31.2%) is retained in the first component (Figure 2A), suggesting only a minor effect of the burial condition on the overall proteome preservation. The 10 proteins with highest contribution to the clustering are plotted in Figure 2B.

**Figure 3:**
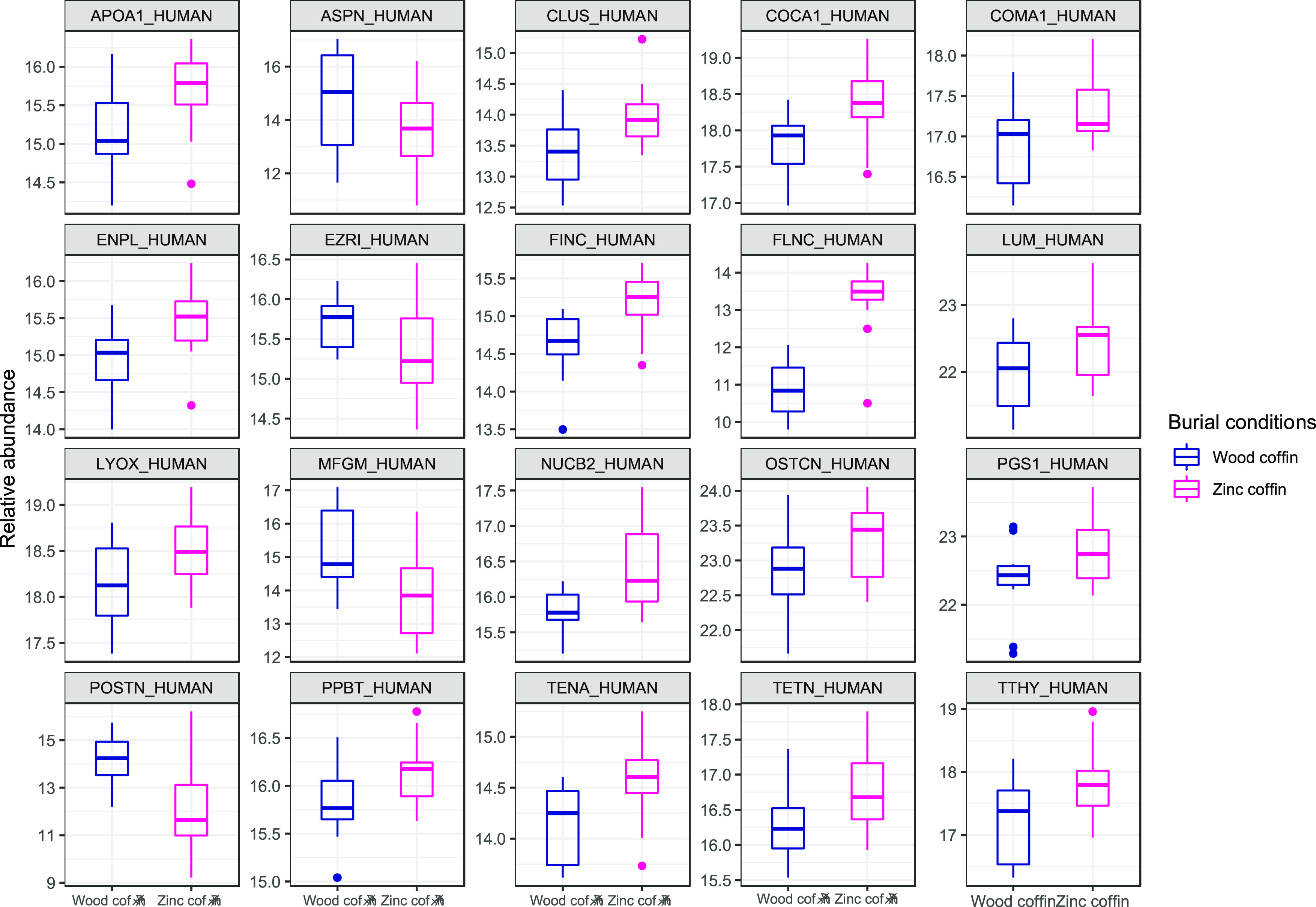
Box plots showing the relative abundances of the 20 proteins that differed significantly (*p* < 0.05) between burial conditions. In blue (first boxes) are samples in the wooden coffin, and in magenta (second boxes) are samples in the zinc-lined coffin (reported as “zinc coffin” in the figure). Supplementary Table S1 provides the entire list of significant proteins according to the *t*-test.

**Figure 4:**
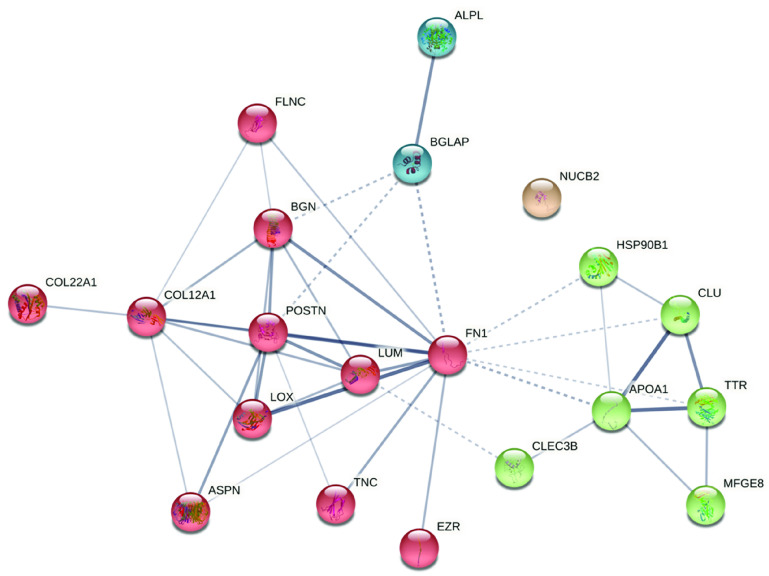
STRING diagram of the proteins that shows the significant difference between the two burial conditions. The clusters show the presence of bone proteins (blue), collagen and collagen-binding proteins (red), also involved in bone metabolism, and plasma and blood proteins (green). NUCB2 is the only protein dissociated from the group. The line thickness indicates the strength of data support (edge confidence). *K* means clustering was used to visualize clusters (*k* = 4).

Despite the inconclusive result of the PCA, *t*-test using the burial condition as the grouping variable allowed for the identification of 20 NCPs whose relative abundances are significantly different between the two groups (Figure 3 and Supplementary Table S1). Sixteen out of 20 NCPs are more abundant in the entombed condition than in the inhumed group. Among the ones more abundant in the inhumed group, we found asporin (ASPN), lactadherin (MFGM), periostin (POSTN), and ezrin (EZRI). ASPN binds calcium and plays a role in osteoblast-driven collagen biomineralization activity. MFGM is responsible for VEGF-dependent neovascularization and the positive regulation of phagocytosis. POSTN is a bone protein involved in tissue development and regeneration as well as extracellular matrix regeneration with the molecular function of a metal-ion-binding protein. Finally, EZRI is a ubiquitous protein not directly involved in bone metabolism. Among proteins more abundant in zinc-lined coffins, there are two proteins associated with the alpha-1 chain of collagen (collagen alpha-1(XXII) chain (COMA1) and collagen alpha-1(XII) chain (COCA1)) and several proteins involved in collagen fibril assembly, organization, binding, and cross-linking (e.g., lumican (LUM), biglycan (PGS1), and fibronectin (FINC)). The same results are also found for calcium-ion-binding proteins, which are involved in bone mineralization and growth and in the control of osteoclasts and osteoblasts (e.g., endoplasmin (ENPL), tetranectin (TETN), tenascin (TENA), osteocalcin (OSTCN), alkaline phosphatase (PPBT), and protein-lysine 6-oxidase (LYOX)). This matches the slightly higher, yet not significant, mean value of C/P found in FTIR for bone buried in a zinc-lined coffin. Individuals buried in a zinc-lined coffin also show a higher relative abundance of clusterin (CLUS), a protein that under conditions of cellular stress promotes the apoptosis of filamin-C (FLNC), a muscle protein involved in myogenesis and structural integrity in muscle fibers, nucleobindin-2 (NUCB2), which is fundamental for calcium homeostasis, transthyretin (TTHY), which is responsible for extracellular matrix organization, and apolipoprotein A-I (APOA1), a transporter serum protein. Figure 4 shows the STRING clustering between different groups of proteins according to the *k*-means algorithm.

### Effect of Burial Conditions on Peptide Deamidation

**Figure 5:**
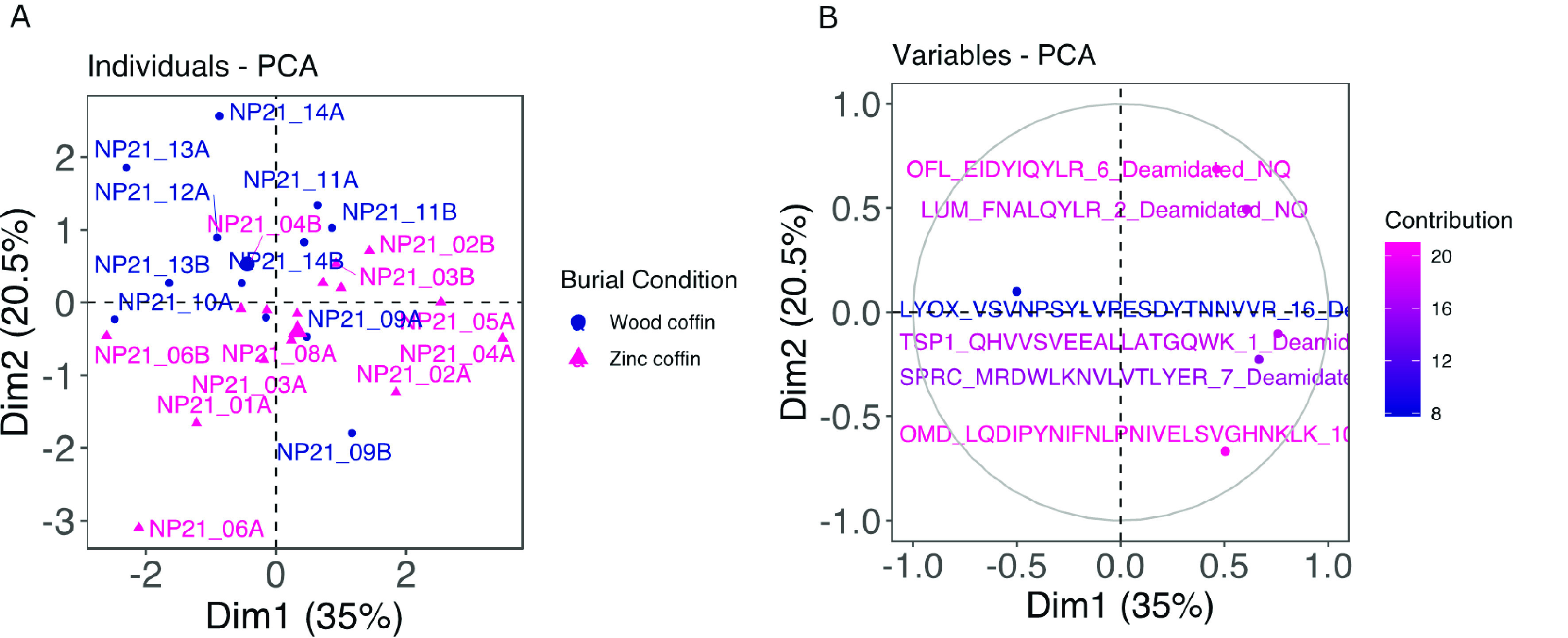
(A) PCA individuals plot showing the clustering according to the two burial conditions, wooden (in blue) and zinc-lined (in magenta) coffins (reported, respectively, as “wood coffin” and “zinc coffin” in the figure), and (B) the contribution of the six deamidated peptides to the PCA.

The other aspect considered in this study in relation to the burial environment is the evaluation of the degree of peptide deamidations. Six peptide sequences are found to have intensities different from zero for all of the specimens under investigation; therefore, deamidation percentages are calculated for each of them (Supplementary Data S1). When tested by PCA (Figure 5A,B), the total variance explained by the model with all six peptides is 55.5%, with no clear clustering between the two groups. In contrast with what is seen for protein relative abundances, here NP21_09 and NP21_10 appear to cluster closer to the wooden coffin group despite belonging to the zinc-lined coffin group.

**Figure 6:**
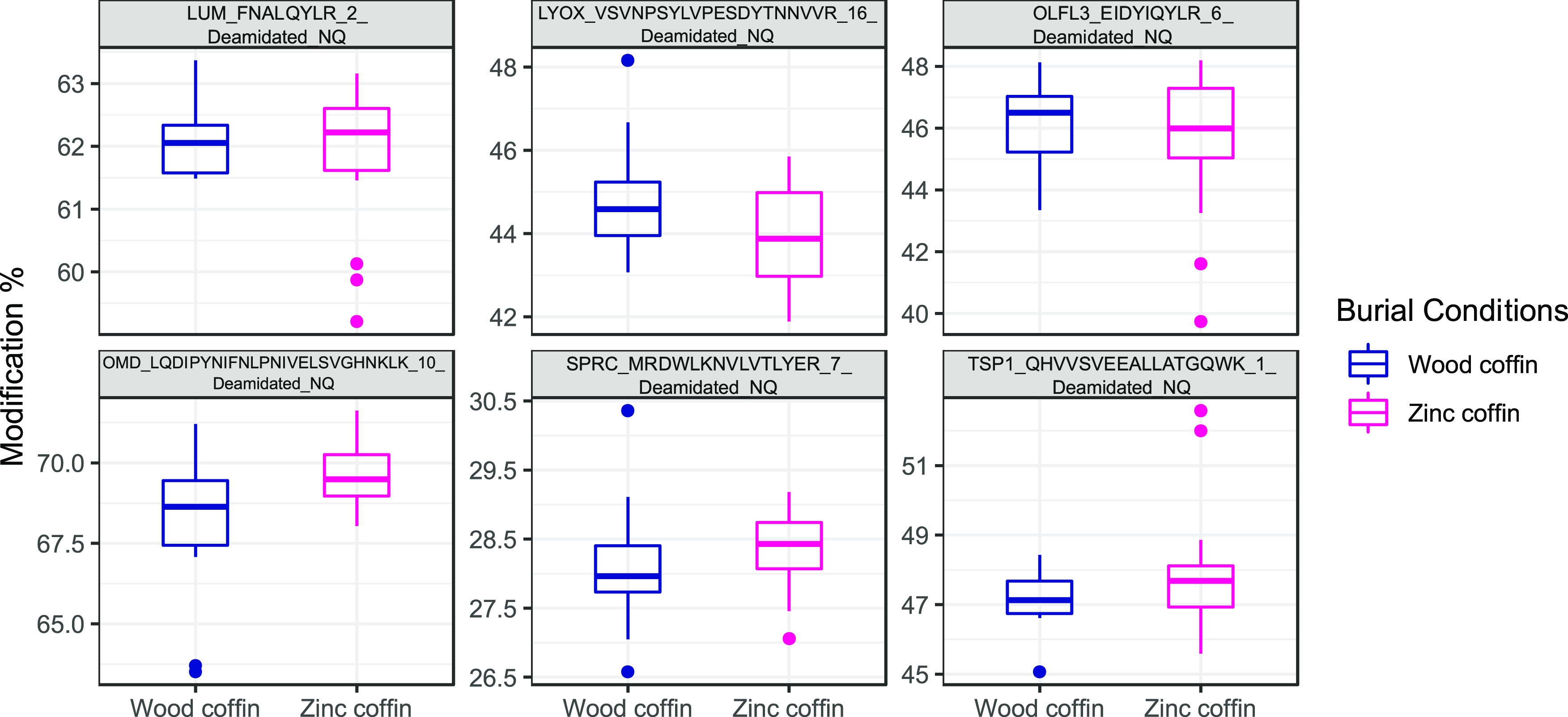
Boxplots showing the deamidation rates for specific peptides and associated proteins (in the title of each boxplot) in wooden (in blue) and zinc-lined (in magenta) coffins (reported, respectively, as “wood coffin” and “zinc coffin” in the figure). No significant differences were found between any of the unmodified and modified peptides.

When comparing mean values for each deamidated peptide (Figure 6), no significant differences are found with the *t*-test between unmodified and modified peptides. However, there is a qualitatively higher deamidation rate, but nonsignificant, for four out of six peptides from the zinc-lined coffin group. These peptides originated, respectively, from osteomodulin (OMD), LUM, SPARC (SPRC), and thrombospondin-1 (TSP1) proteins. Only one peptide behaves in the opposite way, and it matches protein-lysine 6-oxidase (LYOX) (Figure 6). For olfactomedin-like protein 3 (OLFL3), it is not possible to appreciate a qualitative difference between the two groups. OMD is involved in biomineralization processes and binds osteoblasts via the alpha(V)beta3-integrin. LUM is a collagen-binding protein and an extracellular matrix structural constituent known for conferring compression resistance. SPRC is a collagen-binding protein responsible for peptide cross-linking. OLFL3 and TSP1, the peptides showing very similar deamidation levels in both groups, are other extracellular matrix structural constituents. Finally, LYOX is a copper-binding protein involved in collagen fiber organization and bone mineralization.

### Age- and PMI-Related Changes in the Human Proteome

**Figure 7:**
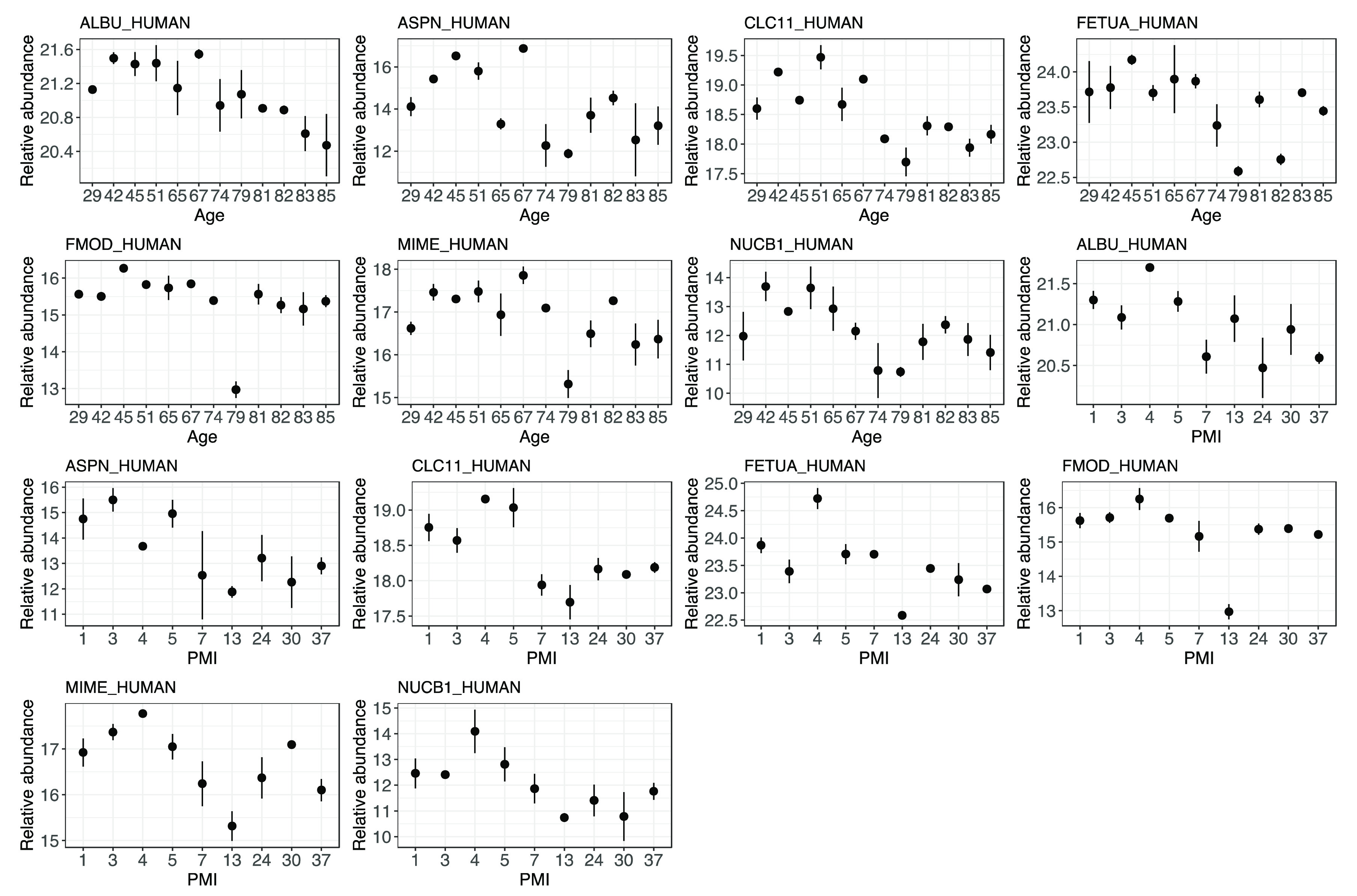
Error plots of the protein abundances showing a significant negative correlation with both age and PMI when *n* = 28 samples are included in the calculation. The first seven plots show the relationship with age, whereas the second seven plots show the correlation of the same protein relative abundances with PMI. Correlation coefficients and significance can be found in Supplementary Table S2.

The last step of data analysis includes correlation analyses to evaluate changes in relative protein abundances and degrees of deamidation (expressed in percentages) and their associations with age or PMI. Among the list of identified proteins, several showed a moderate significant correlation with both age and PMI (Supplementary Table S2), and the error plots for these significant proteins are shown in Figure 7. Albumin (ALBU), for example, has an *R* value of −0.50 with PMI and −0.59 with age. This protein is the primary calcium and magnesium transporter in plasma and also has a shared binding site between zinc and calcium at residue Asp-273 that supports a crosstalk between zinc and calcium transport in the blood. ASPN, as previously mentioned, binds calcium ions and collagen and plays a role in biomineralization. It correlates negatively with both age and PMI (*R* values of, respectively, −0.41 and −0.56). C-type lectin domain family 11 member A (CLC11), a promoter of osteogenesis, is also negatively correlated with PMI (*R* = −0.47). Alpha-2-HS-glycoprotein (FETUA) has an affinity for calcium and barium ions and regulates bone mineralization and negatively correlates with both age and PMI (respectively, *R* = −0.39 and *R* = −0.44). A similar moderate negative correlation was detected for fibromodulin (FMOD, *R* = −0.39 and *R* = −0.40) and mimecan (MIME, *R* = −0.39 and *R* = −0.40), both involved in keratan metabolism. The last protein correlated with both PMI and age is nucleobindin-1 (NUCB1), a calcium-binding protein of the Golgi, which may have a role in calcium homeostasis (*R* = −0.47 and *R* = −0.44).

Considering the proteins correlated exclusively with PMI, it is possible to observe a trend of negative relationships between specific protein abundances and PMIs (Supplementary Figure S1 and Supplementary Table S2) despite the fact that some weak positive relationships are identified for chondroadherin (CHAD) (*R* = 0.38), a protein involved in bone development and in the negative regulation of bone trabeculae formation, for ALBU (*R* = 0.39), and for antithrombin-III (ANT3), which is responsible for the regulation of the blood coagulation cascade. All of the remaining proteins show a significant negative correlation with PMI, respectively, two blood and serum proteins (beta-2-microglobulin (B2MG) and kininogen-1 (KNG1)), a collagen-binding protein (procollagen C-endopeptidase enhancer 1 (PCOC1)), a proteoglycan core protein (basement membrane-specific heparan sulfate proteoglycan core protein (PGMB)), and four ubiquitous proteins (versican core protein (CSPG2), glyceraldehyde-3-phosphate dehydrogenase (G3P), immunoglobulin lambda-like polypeptide 1 (IGL1), and reticulocalbin-3 (RCN3)). Overall, these 10 proteins represent interesting biomarkers for PMI estimation that should be further explored.

**Figure 8:**
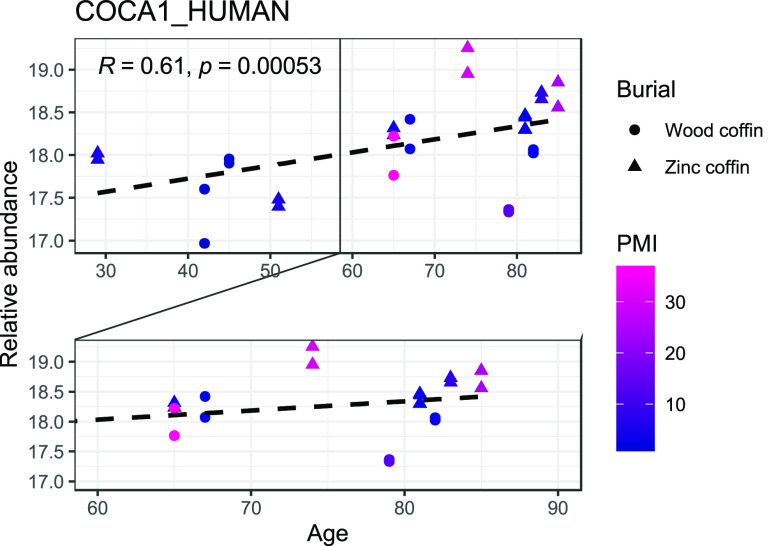
Scatterplot showing the relationship between the relative abundance of COCA1 and the chronological age of the individuals. PMIs are reported using the color code shown in the legend (blue for shorter PMIs, magenta for longer PMIs) and burial conditions (wooden and zinc-lined coffins, reported, respectively, as “wood coffin” and “zinc coffin” in the figure) with different shapes.

When looking at the protein abundances correlated with AAD only, the majority of those show a negative relationship, besides COCA1 (Supplementary Figure S2 and Table S2). Six of these proteins are ubiquitous (amyloid-β precursor protein (A4), complement factor B (CFAB), EZRI, MFGM, protein disulfide-isomerase (PDIA1), and pigment epithelium-derived factor (PEDF)), three are collagenous or collagen-linked (collagen alpha-2(XI) chain (COBA2), COCA1, and COMA1), two are membrane proteins (complement component C9 (CO9) and NUCB1), two are bone proteins (kazal-type serine protease inhibitor domain-containing protein 1 (KAZD1) and OMD), and one is a blood protein (vitamin-K-dependent protein C (PROC)). Among those, the one that requires a specific mention is COCA1, which has a positive relationship with the highest correlation coefficient (Figure 8). It must be noted that older individuals with long PMIs in this sample were predominantly found in zinc-lined coffins (Figure 8); therefore, a “protective” effect of the burial environment on the survival of this protein cannot be fully excluded.

We further correlated the percentage of deamidations with PMI and age (Table 2). The degree of deamidation is not significantly correlated with PMI. However, an accumulation of deamidated residues on the FNALQYLR sequence of LUM is positively correlated with age (*R* = 0.68), suggesting a physiological accumulation of this modification that is minimally affected by PMI. Similarly, a positive weak correlation is also found for SPRC (*R* = 0.42) and OMD (*R* = 0.38) peptides.

**Table 2:** Correlation Coefficients and Significance among the Percentages of Deamidation, PMI, and Chronological Age^a^

deamidation (%)	PMI (years)	age (years)
LUM_F**N**ALQYLR	–0.18	0.68****
SPRC_MRDWLK**N**VLVTLYER	–0.11	0.42*
OLFL3_EIDYI**Q**YLR	–0.26	0.28
LYOX_VSVNPSYLVPESDYT**N**NVVR	0.25	–0.23
OMD_LQDIPYNIF**N**LPNIVELSVGHNKLK	0.24	0.38*
TSP1_**Q**HVVSVEEALLATGQWK	–0.16	0.34

a Three proteins show a positive significant correlation (*p<0.05; ****p<0.0001) with age, whereas none show a positive correlation with PMI. Deamidated asparagine (N) and glutamine (Q) residues are in bold.

## Discussion

Different burial environments affect the preservation of the bone proteomes in different ways, and this could have an impact on the estimation of the PMI and AAD in forensic contexts. The present study employs LC-MS/MS to evaluate the survival of proteins and the variation in PTMs of femoral cortical bone from 14 individuals buried in either zinc-lined or wooden coffins at different PMI intervals. It also provides FTIR results to test the presence of variations associated with the bone matrix composition that could be reflected in changes in the relative abundances of NCPs. PCA showed partial clusters when evaluating protein abundances or PTM percentages, with a total variance explained ranging between ∼41 and ∼55%, respectively. When considering *t*-test results, only 4 proteins showed higher abundances for the inhumed group, whereas the remaining 16 were better preserved in the entombed group. The results also showed that there are not significant differences in the deamidation percentages for the two burial conditions. Finally, several proteins showed relationships with the PMI and age, with correlation coefficients of up to 0.69 for AAD for vitamin-K-dependent protein C and 0.68 for PMI for versican core protein. Overall, this study shows the potential of the proteomic approach in forensic investigations, not only to better understand post-mortem phenomena and their connection to biomolecule preservation and bioerosion but also to reveal additional potential biomarkers for PMI and AAD estimation.

### Implication of Different Burial Environments on Protein Survival

PCA calculated using the relative abundances for all of the proteins extracted from the human femora revealed only a partial clustering of the samples according to their burial conditions (Figure 2A). The sample type used in the study has noteworthy variation in terms of both the AAD (29–85 years) and the PMI (1–37 years), and the individuals are not equally distributed across age or PMI. In particular, for PMI, seven individuals between the two groups have a PMI of <5 years, three are between 5 and 10, one is between 10 and 20, one is between 20 and 30 years, and the remaining have a PMI of 30+ years. Considering these two factors, the modest sample size (*n* = 14), and the potential intrinsic factors (i.e., body mass, cause of death) that could affect protein degradation, it is not surprising that PCA did not show a sharp separation between the two burial conditions and that the dimensionality reduction approach was not able to comprehensively explain the influence of different environments on the proteomes. The lack of details regarding the specific environmental conditions to which each body has been subjected further limits our understanding of the post-mortem survivability of the bone proteome in this precise sample, as these factors are known to play a crucial role in protein survival.^40^ One aspect highlighted by Guareschi et al.^8^ is the practice of breaching zinc coffins to facilitate the decomposition. This can play a central role in the rate of decay and therefore protein survival, as it exposes the corpse to exogenous bacteria.^20^ Decomposition would seem to be promoted in a structurally compromised coffin in comparison with intact stone tombs, for which only ∼11% of the individuals were found completely skeletonized after the first exhumation.^8^ They also found that lined coffins offer a more chemically stable environment to the body, limiting the microbiological action and resulting in considerably slower decomposition rates.^20^ In our study, all wooden coffins were found to be structurally compromised upon excavation, whereas all zinc-lined coffins were still intact. For this reason, it was not unexpected that most of the proteins survived better under the latter conditions than under the former conditions. We found 16 out of 20 proteins to be more abundant in the entombed cohort in comparison with the inhumed cohort (Figure 3). Among these 16 proteins, we found a set of collagenous, collagen-binding, and bone-specific proteins, such as two collagen chains (alpha-1(XXII) and alpha-1(XII)), biglycan, lumican, fibronectin, osteocalcin, alkaline phosphatase, and tetranectin, as well as plasma and muscle proteins, such as apolipoprotein A-I, clusterin, filamin-C, nucleobindin-2, and transthyretin. Their improved preservation may be attributed to the protective function of the zinc lining that creates a relatively stable anaerobic environment around the cadaver, preventing the access and the action of exogenous bacteria.^41^ Furthermore, zinc-lined coffins are longer lasting compared with wooden coffins, and this condition limits severe humidity and temperature changes, providing a more chemically and microbiologically stable environment than the inhumed conditions.^34^ It has been previously shown that bioerosion significantly impacts the survival of bone proteins;^34^ however, the contribution that either endogenous and exogenous bacterial communities have on bones and which one was responsible for the observed protein degradation are still not clear.^42,43^ This study shows that the exogenous microbial populations, together with environmental and taphonomic factors, play a key role in bone protein survival and therefore that the decay effect caused by the action of the gut bacteria alone does not resemble that observed in the inhumed cadavers. The study conducted at La Villetta cemetery in Parma (Italy) showed that the formation of adipocere and the limited cadaveric decomposition are facilitated by the entombment, in particular, when the coffins have not been breached to accelerate the decomposition process.^8^ Our results may therefore support, from a biomolecular point of view, what was previously observed by Guareschi et al.,^8^ and therefore that the entombment condition allows for a better preservation of soft tissues and bones and therefore an overall better survival of bone proteins in these circumstances.

An unexpected outcome is represented by the four proteins that show an increased abundance under the inhumed conditions (Figure 3). One of those is lactadherin, a protein that positively regulates osteoblastogenesis and osteoblast activity and suppresses the osteoclast activity. As a result, the repression of this gene is associated with bone loss caused by the increase in bone resorption^44^ associated with aging. The higher abundance of lactadherin observed for the individuals buried in wooden coffins therefore may be associated with the younger average age of these individuals rather than with the deposition type, as also proven by the correlation coefficient between this protein and the AAD (Supplementary Table S2). A similar trend is found for asporin, with a higher relative abundance in the inhumed cadavers than in the other group. Asporin is involved in cartilage and bone degeneration and plays a role in the insurgence of osteoarthritis.^45^ Similarly to lactadherin, also in this case, there is a significant negative correlation between its abundance and age (Supplementary Table S2); therefore, for the same reason previously explained, the notion that the deposition type caused the observed differences in the relative abundances among the two groups can be excluded. Finally, a similar trend was also observed for ezrin and periostin. Ezrin is a protein with a role in calcium homeostasis.^46^ Periostin plays a central role in osteogenesis and response to mechanical stress, and it is involved in the proteolytic activation of lysyl oxidase, which leads to the formation of collagen cross-linking.^47,48^ The reduced production of periostin with aging could be one of the phenomena responsible for the accumulation of microdamage in bones and for the overall increase in bone fragility in elderly individuals.^49,50^ Whereas we found a strong correlation between ezrin and the AAD (Supplementary Table S2), we did not detect a statistical correlation between periostin and age in this study. Taking all of these evidences into consideration, we believe that the results observed for these four proteins may have been biased by the fact these are negatively correlated with age. Considering, in fact, that the average age for the inhumed group is slightly lower than that for the entombed group (63.33 ± 16.74 versus 68.62 ± 19.65), that the first group is characterized by six individuals whereas the second group is characterized by eight, and that the individuals with the highest abundances of these proteins were two specific ones out of these six (respectively, NP21_12A/B, aged 45, and NP21_14A/B, aged 67), we cannot exclude the possibility that these proteins were already more abundant in these individuals at the time of death.

On the contrary, among the 16 proteins more abundant in the entombed cadavers, only two are significantly correlated with AAD and one with PMI, therefore excluding the presence of potential “biases” associated with either the AAD or PMI in this group. It is worth highlighting that the four proteins previously highlighted should be further investigated from a physicochemical perspective in future studies to better explain this behavior and to eventually test whether their decay will be similar in entombed versus inhumed cadavers sharing a similar AAD.

### Deamidation and Burial Environment

The increase in deamidation rates has been seen to be associated with burial conditions in both terrestrial and aquatic environments. van Doorn et al.,^51^ studying 911 bone collagen samples from 50 archeological sites, showed that the amount of glutamine deamidation was highly dependent on the burial conditions and on the thermal age. This is similar in aquatic environments, where nonenzymatic deamidations increase with increasing post-mortem submerged intervals in different ways for different types of water.^3^ Considering the results found here, we believe that the interaction among the body, body fluids, and the zinc environment may be one of the causes for the observed differences in deamidations rates. In particular, the entombed status can be considered as a “closed system”, where different parameters, such as the lack of oxygen, cannot be altered by exogenous factors. Contrariwise, the inhumed status, regarded as an “open system”, represents a less controllable scenario because of the immediate access to the soil surrounding the coffin. In these circumstances, the complex interaction between both intrinsic and extrinsic variables plays a role in determining the chemical modifications affecting proteins that are difficult to be controlled and predicted. In this study, PCA calculated on the deamidation percentages of six peptides shows a better separation between the two burial conditions (explaining 55.5% of the total variance with the first two components) when compared with the protein abundances. The only two samples that seem to cluster with the wrong group are NP21_9 (13 years PMI) and NP21_10 (37 years PMI), both buried in wooden coffins. NP21_10, after deposition in the wooden coffin for an unknown period of time, was deposited in a marble burial cell wrapped in a cotton sheet, as it was partially mummified; therefore, it could have had a slower decomposition process than the others. This observation supports once more the hypothesis that wooden coffins are “open systems” with a persisting, although limited, amount of oxygen able to support the mummification of the body. This may explain why the PTMs in NP21_10 resulted in being more similar to those found in the entombed environment than those found in the inhumed environment. For NP21_9, there are no obvious observations for the cadaver that may explain this behavior; there may have been specific ante-mortem conditions able to have influenced the resulting PTMs, but the lack of ante-mortem data does not allow us to clearly understand the reasons behind this finding. It has to be noted that despite the promising results obtained with the PCA, there were no peptides with a significant difference in deamidation ratios between the two groups (Figure 6). This result suggests that peptide PTMs may not be the ideal target for conducting these comparative analyses between different burial conditions on relatively short forensic time scales, as they may be less prone to variations caused by different post-mortem environments than the proteome variety and abundance. It is not possible to exclude, as indicated previously, that other factors able to affect PTMs may be associated with the physicochemical and structural properties of the proteins as well as with both interskeletal and interindividual variability.^3,5^ Therefore, more systematic models should be created to fully untangle the biochemistry on the accumulation of PTMs in different burial environments.

### PMI- and Age-Related Changes in Proteome and Deamidation

In this specific study, we also evaluated the presence of correlation trends with AAD and PMI in the entire sample. When considering protein abundances, PMI, and AAD, we identified three main behaviors for the proteins significantly correlated with these variables: (I) a constant decrease, (II) a sudden drop at a certain time in the PMI or AAD, and (III) a mild decrease with outliers. Before proceeding with the discussion of each of these behaviors, it has to be considered that this study, due to the nature of the material (forensic cases), lacks sample standardization for potential influencing factors (i.e., chronological age, PMIs, ante-mortem condition, and case-to-case environmental differences). Therefore, the following arguments have been hypothesized and discussed, considering previously published findings, and have to be tested in a more standardized and controlled manner before these findings can be considered conclusive.

(I) Albumin shows a very stable and consistent decrease in relative abundance with both the PMI and AAD. This protein is one of the most abundant in bone,^17^ and it has been previously reported to survive for long periods of time, as it has been found in archeological materials up to 4000 years old.^52^ A recent study showed that albumin is negatively correlated with AAD in tibia samples, despite the fact that the results were not statistically significant.^5^ The constant degradation of albumin with increasing PMI and its lower relative abundance in elderly individuals make it an ideal marker for forensic estimations. Similarly, fetuin-A, a serum protein secreted by the liver and involved in bone mineralization, has a negative correlation with both the PMI and age. Considering the fetuin-A levels and the AAD, the results found here agree with what was found by Procopio et al.^4^ on pigs, Sawafuji et al.^53^ on human archeological samples, and Mickleburgh et al.^5^ on modern human samples. When focusing on the fetuin-A levels and PMI, the results are partially in contrast with what was found by Procopio et al.^17^ using pig bones, where no significant variations in fetuin-A abundances were found up to a PMI of 6 months. Fetuin-A could therefore be stable over short PMIs and start to decrease with prolonged PMIs; for this reason, employing fetuin-A for AAD estimation may be applicable only for cadavers with relatively short PMIs. Both of these proteins offer the advantage of being minimally affected by the burial environment, excluding biases in the estimations. It must be highlighted that both of these proteins were previously proposed as potential biomarkers for AAD and PMI in both animal and in human studies;^17,18^ here, despite the limited sample size and its nonideal distribution among different PMIs and AADs, we were able to confirm their usefulness and significance for aging estimations in forensic contexts.

(II) Asporin, C-type lectin, and mimecan show a considerable drop in their relative abundances at a PMI of 5 years and at ∼70 years of age. These proteins are not directly associated with bone metabolism and do not have the same affinity for hydroxyapatite (HAp) as the previously mentioned albumin and fetuin-A; therefore, they are more likely to survive in bones for shorter periods of times. Surprisingly, according to our results, these proteins are not affected by the burial environment.^34^

(III) Fibromodulin, a small leucine-rich proteoglycan that has a strong affinity for the HAp matrix,^54^ shows a minor decrease associated with age and PMI, and its correlation coefficients may have been significantly improved if the outlier NP21_09A/B, which has a significantly lower abundance than most of the proteins highlighted here, had been excluded. This issue is likely linked to an intrinsic biological variability that characterizes this individual with respect to others, such as the presence of a specific pathological condition, environmental factors, or merely interindividual biological variability.^4^

Regarding the proteins correlated with PMI only (Supplementary Figure S1), eight proteins show a negative correlation, and only two proteins show a positive correlation (antithrombin-III and chondroadherin). Although no clear explanations can be provided for the proteins whose abundances increases over time, the low and variable number of individuals for each point in the PMI does not allow us to reach any conclusion. As expected, most of the proteins show a negative relationship with the PMI. Versican core protein, a chondroitin sulfate proteoglycan involved in woven bone formation and bone development,^55^ shows the most consistent degradation over the course of time. The longevity of this class of proteins has been previously confirmed by the analysis of archeological bones and teeth,^56^ suggesting its potential application in palaeontology, archeology, and forensic sciences. The presence of a single collagen-binding protein in this group, namely, procollagen C-endopeptidase enhancer 1, suggests that NCPs with a high affinity for the mineral matrix may be more resistant to hydrolysis than collagens and collagen-binding proteins. This result further confirms previous findings on the successful recovery of these NCPs in archeological contexts, even when only a minimal amount of intact collagen is available.^57^

Considering the proteins correlated uniquely with AAD (Supplementary Figure S2), the best positive correlation was shown by the collagen alpha-1(XII) chain. The surprising behavior of COCA1 could be linked to the protective role of the zinc lining: In fact, the older individuals present in this study were placed in zinc-lined coffins. This further supports the idea that the burial environment can play a key role in the preservation of both collagenous proteins and NCPs, and this should be taken into account when selecting specific markers for AAD and PMI estimation. The remaining collagenous proteins (COMA1, COBA2) were both negatively affected by age, as expected, and may be good markers for age estimation. All of the other proteins with a correlation coefficient of >0.60 have a bone-specific function associated with their maintenance and turnover (CLC11, OMD, PROC). Their lower abundance in coincidence with the increase in chronological age is associated with the well-known increase in bone fragility,^58^ and their survival with prolonged PMIs is associated with their strong affinity for HAp that confers longevity and also makes them good candidates for AAD estimation after extended PMI.^17,18^ An association between chronological age and specific protein abundances has also been confirmed in muscle tissue. A recent study involving *Musculus vastus lateralis* showed that individuals of ages between 18 and 80 years undergo different protein degradation patterns compared with individuals outside this range, highlighting the importance of age correction factors when developing PMI prediction models.^59^

Surprisingly, deamidation showed a significant positive correlation only with age. The best correlation between age and asparagine deamidation is found in lumican, a proteoglycan that is found in the bone matrix, which is secreted by differentiating osteoblasts.^60^ Degradation of lumican was seen in intervertebral disks with increasing age.^61^ Thus, with aging, the organism is not able to replace the modified proteins with new unmodified proteins, resulting in the accumulation of PTMs.^62,63^ From a more forensic point of view, Procopio et al.^17^ in a study on porcine bones, showed that asparagine deamidation of biglycan, a proteoglycan similar to lumican, is stable for the first 2 month post-mortem and significantly increases across the following 4 months. It is not surprising that the deamidation identified in this study as being associated with chronological age is of asparagine and not of glutamine, as the lifespan of this process is notably quicker than that taking place on glutamine residues, which are indeed more studied in archeological samples than in forensic timeframes. The increase in deamidations is the result of a nonenzymatic process that takes place physiologically both ante-mortem and post-mortem.^17,63^ Further research should focus on the application of this approach in a more comprehensive way by employing a sample that comprises an adequate number of individuals per PMI point and that is balanced in terms of age. Because of the influence of environmental parameters, it would be ideal to record them throughout the entire experiment.

## Conclusions

In the present study, we applied proteomics to a forensic sample made up of 14 human femoral fragments that originated from Italian juridical caseworks to identify the effect that different burial environments (entombed in zinc-lined vs inhumed in wooden coffins) may have on potential proteomic biomarkers for AAD and PMI estimation from skeletal remains. The overall results show that the protected environment offered by the zinc-lined coffin allows for a better preservation of NCPs than the inhumed environment, probably by limiting the bioerosion activity caused by exogenous bacteria. Therefore, we can assume that a different effect is operated by endogenous and exogenous bacteria on the bone proteome; entombed bones are better preserved than inhumed ones because of the sole erosive action of endogenous (gut) bacteria, whereas the combination of endogenous and exogenous bacteria severely impacts the proteome. In terms of PTMs, none of the identified peptides showed a significant difference in deamidation ratio percentages between the two burial conditions. Regarding the identification of potential biomarkers for aging estimations, despite the presence of the two different burial environments, we could highlight NCPs previously reported for their relationship with the AAD and PMI (e.g., fetuin-A and albumin) and new NCPs for AAD estimation (e.g., osteomodulin and vitamin-K-dependent protein C) and PMI estimation (e.g., versican core protein and beta-2-microglobulin) that could be validated and employed in the future for forensic investigations. Despite the preliminary nature of this study, we showed that proteomics could provide biomarkers with little or no bias due to the type of coffin employed in the burial and clarify the role that endogenous and exogenous bacteria play in decomposition and bone degradation. Overall, the study highlights the potential of bone proteomics for forensic application and the importance of evaluating the effect of biasing factors such as the burial environment, sample composition, and biological interindividual variation. Furthermore, the behavior of each protein should be assessed singularly, as different functions and properties might lead to nonlinear relationships with the AAD and PMI. Further research should focus on applying this approach in a more comprehensive way by employing a larger sample homogeneously distributed across the entire age range and PMI.

## Supporting Information Available

Supplementary Table S1. Mean and standard deviation (SD) values for the proteins showing the significant difference between the two burial conditions. Supplementary Table S2. Correlation coefficients between each protein and both age and PMI. Supplementary Figure S1. Error plots of proteins correlating significantly with PMI only. Supplementary Figure S2. Error plots of proteins correlating significantly with age only. (PDF)
Supplementary Data S1. Deamidation percentages for the six peptides highlighted in the study in both zinc-lined (samples 1–8) and wooden (samples 9–14) coffins (XLSX)

## Notes

The mass spectrometry proteomics data have been deposited to the PRIDE Archive (http://www.ebi.ac.uk/pride/archive/) via the PRIDE partner repository with the data set identifier PXD029911 and 10.6019/PXD029911.

## References

[ref1] Cockle D. L.; Bell L. S. Human Decomposition and the Reliability of a “Universal” Model for Post Mortem Interval Estimations. Forensic Science International 2015, 253, 136.e1–136.e9. 10.1016/j.forsciint.2015.05.018. 26092190

[ref2] Taphonomy of Human Remains: Forensic Analysis of the Dead and the Depositional Environment, 1st ed.; Schotsmans E. M. J., Márquez-Grant N., Forbes S. L., Eds.; John Wiley & Sons, Ltd: Chichester, U.K., 2017.

[ref3] Mizukami H.; Hathway B.; Procopio N. Aquatic Decomposition of Mammalian Corpses: A Forensic Proteomic Approach. J. Proteome Res. 2020, 19 (5), 2122–2135. 10.1021/acs.jproteome.0c00060. 32242669

[ref4] Procopio N.; Chamberlain A. T.; Buckley M. Intra- and Interskeletal Proteome Variations in Fresh and Buried Bones. J. Proteome Res. 2017, 16 (5), 2016–2029. 10.1021/acs.jproteome.6b01070. 28436665

[ref5] Mickleburgh H. L.; Schwalbe E. C.; Bonicelli A.; Mizukami H.; Sellitto F.; Starace S.; Wescott D. J.; Carter D. O.; Procopio N. Human Bone Proteomes before and after Decomposition: Investigating the Effects of Biological Variation and Taphonomic Alteration on Bone Protein Profiles and the Implications for Forensic Proteomics. J. Proteome Res. 2021, 20 (5), 2533–2546. 10.1021/acs.jproteome.0c00992. 33683123 PMC8155572

[ref6] Jopp-Van Well E.; Augustin C.; Busse B.; Fuhrmann A.; Hahn M.; Tsokos M.; Verhoff M.; Schulz F. The Assessment of Adipocere to Estimate the Post-Mortem Interval – a Skeleton from the Tidelands. Anthropologischer Anzeiger 2016, 73 (3), 235 10.1127/anthranz/2016/0615. 27189778

[ref7] Ubelaker D. H.; Zarenko K. M. Adipocere: What Is Known after over Two Centuries of Research. Forensic Science International 2011, 208 (1–3), 167–172. 10.1016/j.forsciint.2010.11.024. 21185137

[ref8] Guareschi E.; Dadour I. R.; Magni P. A. A Taphonomic Examination of Inhumed and Entombed Remains in Parma Cemeteries, Italy. Global Journal of Forensic Science & Medicine 2019, 1 (4), GJFSM.MS.ID.000518 10.33552/GJFSM.2019.01.000518.

[ref9] Fielder M.; Nair A. K. Effects of Hydration and Mineralization on the Deformation Mechanisms of Collagen Fibrils in Bone at the Nanoscale. Biomechanics and Modeling in Mechanobiology 2019, 18 (1), 57–68. 10.1007/s10237-018-1067-y. 30088113

[ref10] Woess C.; Unterberger S. H.; Roider C.; Ritsch-Marte M.; Pemberger N.; Cemper-Kiesslich J.; Hatzer-Grubwieser P.; Parson W.; Pallua J. D. Assessing Various Infrared (IR) Microscopic Imaging Techniques for Post-Mortem Interval Evaluation of Human Skeletal Remains. PLoS One 2017, 12 (3), e0174552 10.1371/journal.pone.0174552. 28334006 PMC5363948

[ref11] Creagh D.; Cameron A. Estimating the Post-Mortem Interval of Skeletonized Remains: The Use of Infrared Spectroscopy and Raman Spectro-Microscopy. Radiat. Phys. Chem. 2017, 137, 225 10.1016/j.radphyschem.2016.03.007.

[ref12] Longato S.; Wöss C.; Hatzer-Grubwieser P.; Bauer C.; Parson W.; Unterberger S. H.; Kuhn V.; Pemberger N.; Pallua A. K.; Recheis W.; Lackner R.; Stalder R.; Pallua J. D. Post-Mortem Interval Estimation of Human Skeletal Remains by Micro-Computed Tomography, Mid-Infrared Microscopic Imaging and Energy Dispersive X-Ray Mapping. Analytical Methods 2015, 7 (7), 2917–2927. 10.1039/C4AY02943G. 25878731 PMC4383336

[ref13] Amadasi A.; Cappella A.; Cattaneo C.; Cofrancesco P.; Cucca L.; Merli D.; Milanese C.; Pinto A.; Profumo A.; Scarpulla V.; Sguazza E. Determination of the Post Mortem Interval in Skeletal Remains by the Comparative Use of Different Physico-Chemical Methods: Are They Reliable as an Alternative to 14C?. HOMO 2017, 68 (3), 213 10.1016/j.jchb.2017.03.006. 28404240

[ref14] Parker G. J.; McKiernan H. E.; Legg K. M.; Goecker Z. C. Forensic Proteomics. Forensic Science International: Genetics 2021, 54, 102529 10.1016/j.fsigen.2021.102529. 34139528

[ref15] Duong V.-A.; Park J.-M.; Lim H.-J.; Lee H. Proteomics in Forensic Analysis: Applications for Human Samples. Applied Science 2021, 11 (8), 3393 10.3390/app11083393.

[ref16] Choi K. M.; Zissler A.; Kim E.; Ehrenfellner B.; Cho E.; Lee S. in; Steinbacher P.; Yun K. N.; Shin J. H.; Kim J. Y.; Stoiber W.; Chung H.; Monticelli F. C.; Kim J. Y.; Pittner S. Postmortem Proteomics to Discover Biomarkers for Forensic PMI Estimation. International Journal of Legal Medicine 2019, 133 (3), 899–908. 10.1007/s00414-019-02011-6. 30864069 PMC6469664

[ref17] Procopio N.; Williams A.; Chamberlain A. T.; Buckley M. Forensic Proteomics for the Evaluation of the Post-Mortem Decay in Bones. Journal of Proteomics 2018, 177, 21–30. 10.1016/j.jprot.2018.01.016. 29407476

[ref18] Prieto-Bonete G.; Pérez-Cárceles M. D.; Maurandi-López A.; Pérez-Martínez C.; Luna A. Association between Protein Profile and Postmortem Interval in Human Bone Remains. Journal of Proteomics 2019, 192, 54–63. 10.1016/j.jprot.2018.08.008. 30145274

[ref19] Forbes S. L.; Stuart B. H.; Dent B. B. The Effect of the Method of Burial on Adipocere Formation. Forensic Science International 2005, 154 (1), 44–52. 10.1016/j.forsciint.2004.09.109. 16182948

[ref20] Goff M. L. Early Post-Mortem Changes and Stages of Decomposition in Exposed Cadavers. Experimental and Applied Acarology 2009, 49 (1–2), 21–36. 10.1007/s10493-009-9284-9. 19554461

[ref21] Javan G. T.; Finley S. J.; Can I.; Wilkinson J. E.; Hanson J. D.; Tarone A. M. Human Thanatomicrobiome Succession and Time since Death. Sci. Rep. 2016, 6, 29598 10.1038/srep29598. 27412051 PMC4944132

[ref22] Jans M. M. E.; Nielsen-Marsh C. M.; Smith C. I.; Collins M. J.; Kars H. Characterisation of Microbial Attack on Archaeological Bone. Journal of Archaeological Science 2004, 31 (1), 87–95. 10.1016/j.jas.2003.07.007.

[ref23] Melvin J. R.; Cronholm L. S.; Simson L. R.; Isaacs A. M. Bacterial Transmigration as an Indicator of Time of Death. Journal of Forensic Sciences 1984, 29 (2), 11687J 10.1520/JFS11687J. 6726153

[ref24] Kellerma G. D.; Waterman N. G.; Scharfenberger L. F. Demonstration in Vitro of Postmortem Bacterial Transmigration. American Journal of Clinical Pathology 1976, 66 (5), 911 10.1093/ajcp/66.5.911. 790938

[ref25] Jans M. M. E. Microbial Bioerosion of Bone - A Review. In Current Developments in Bioerosion; Wisshak M., Tapanila L., Eds.; Springer: Berlin, 2008; pp 397–413.

[ref26] Hyde E. R.; Haarmann D. P.; Lynne A. M.; Bucheli S. R.; Petrosino J. F. The Living Dead: Bacterial Community Structure of a Cadaver at the Onset and End of the Bloat Stage of Decomposition. PloS one 2013, 8 (10), e77733 10.1371/journal.pone.0077733. 24204941 PMC3813760

[ref27] Finley S. J.; Benbow M. E.; Javan G. T. Microbial Communities Associated with Human Decomposition and Their Potential Use as Postmortem Clocks. International Journal of Legal Medicine 2015, 129 (3), 623–632. 10.1007/s00414-014-1059-0. 25129823

[ref28] Damann F. E.; Jans M. M. E. Microbes, Anthropology, and Bones. In Forensic Microbiology; Carter D. O., Tomberlin J. K., Benbow M. E., Metcalf J. L., Eds.; John Wiley & Sons: West Sussex, UK, 2017; pp 312–327.

[ref29] Hyde E. R.; Haarmann D. P.; Petrosino J. F.; Lynne A. M.; Bucheli S. R. Initial Insights into Bacterial Succession during Human Decomposition. International Journal of Legal Medicine 2015, 129 (3), 661–671. 10.1007/s00414-014-1128-4. 25431049

[ref30] Metcalf J. L.; Wegener Parfrey L.; Gonzalez A.; Lauber C. L.; Knights D.; Ackermann G.; Humphrey G. C.; Gebert M. J.; van Treuren W.; Berg-Lyons D.; Keepers K.; Guo Y.; Bullard J.; Fierer N.; Carter D. O.; Knight R. A Microbial Clock Provides an Accurate Estimate of the Postmortem Interval in a Mouse Model System. eLife 2013, 2, e01104 10.7554/eLife.01104. 24137541 PMC3796315

[ref31] Hyde E. R.; Metcalf J. L.; Bucheli S. R.; Lynne A. M.; Knight R. Microbial Communities Associated with Decomposing Corpses. In Forensic Microbiology; Carter D. O., Tomberlin J. K., Benbow M. E., Metcalf J. L., Eds.; John Wiley & Sons: West Sussex, U.K., 2017; pp 245–273.

[ref32] Metcalf J. L. Estimating the Postmortem Interval Using Microbes: Knowledge Gaps and a Path to Technology Adoption. Forensic Science International: Genetics 2019, 38, 211–218. 10.1016/j.fsigen.2018.11.004. 30448529

[ref33] Procopio N.; Ghignone S.; Voyron S.; Chiapello M.; Williams A.; Chamberlain A.; Mello A.; Buckley M. Soil Fungal Communities Investigated by Metabarcoding Within Simulated Forensic Burial Contexts. Frontiers in Microbiology 2020, 10.3389/fmicb.2020.01686. PMC7393272 32793158

[ref34] Procopio N.; Mein C. A.; Starace S.; Bonicelli A.; Williams A. Bone Diagenesis in Short Timescales: Insights from an Exploratory Proteomic Analysis. Biology 2021, 10 (6), 460 10.3390/biology10060460. 34071025 PMC8224596

[ref35] Kontopoulos I.; Presslee S.; Penkman K.; Collins M. J. Preparation of Bone Powder for FTIR-ATR Analysis: The Particle Size Effect. Vib. Spectrosc. 2018, 99 (August), 167–177. 10.1016/j.vibspec.2018.09.004.

[ref36] Procopio N.; Buckley M. Minimizing Laboratory-Induced Decay in Bone Proteomics. J. Proteome Res. 2017, 16 (2), 447–458. 10.1021/acs.jproteome.6b00564. 28152590

[ref37] Johnson W. E.; Li C.; Rabinovic A. Adjusting Batch Effects in Microarray Expression Data Using Empirical Bayes Methods. Biostatistics 2007, 8 (1), 118–127. 10.1093/biostatistics/kxj037. 16632515

[ref38] Szklarczyk D.; Gable A. L.; Lyon D.; Junge A.; Wyder S.; Huerta-Cepas J.; Simonovic M.; Doncheva N. T.; Morris J. H.; Bork P.; Jensen L. J.; Mering C. von. STRING V11: Protein–Protein Association Networks with Increased Coverage, Supporting Functional Discovery in Genome-Wide Experimental Datasets. Nucleic Acids Res. 2019, 47 (D1), D607–D613. 10.1093/nar/gky1131. 30476243 PMC6323986

[ref39] Wilson J.; van Doorn N. L.; Collins M. J. Assessing the Extent of Bone Degradation Using Glutamine Deamidation in Collagen. Anal. Chem. 2012, 84 (21), 9041–9048. 10.1021/ac301333t. 23030643

[ref40] Zissler A.; Stoiber W.; Geissenberger J.; Steinbacher P.; Monticelli F. C.; Pittner S. Influencing Factors on Postmortem Protein Degradation for Pmi Estimation: A Systematic Review. Diagnostics 2021, 11 (7), 1146 10.3390/diagnostics11071146. 34201836 PMC8304065

[ref41] Dent B. B.; Forbes S. L.; Stuart B. H. Review of Human Decomposition Processes in Soil. Environmental Geology 2004, 45 (4), 576–585. 10.1007/s00254-003-0913-z.

[ref42] Booth T. J. An Investigation Into the Relationship Between Funerary Treatment and Bacterial Bioerosion in European Archaeological Human Bone. Archaeometry 2016, 58 (3), 484–499. 10.1111/arcm.12190.

[ref43] Turner-Walker G. Light at the End of the Tunnels? The Origins of Microbial Bioerosion in Mineralised Collagen. Palaeogeography, Palaeoclimatology, Palaeoecology 2019, 529, 24–38. 10.1016/j.palaeo.2019.05.020.

[ref44] Sinningen K.; Albus E.; Thiele S.; Grossklaus S.; Kurth T.; Udey M. C.; Chavakis T.; Hofbauer L. C.; Rauner M. Loss of Milk Fat Globule-Epidermal Growth Factor 8 (MFG-E8) in Mice Leads to Low Bone Mass and Accelerates Ovariectomy-Associated Bone Loss by Increasing Osteoclastogenesis. Bone 2015, 76, 107–114. 10.1016/j.bone.2015.04.003. 25868798

[ref45] Xu L.; Li Z.; Liu S. Y.; Xu S. Y.; Ni G. X. Asporin and Osteoarthritis. Osteoarthritis and Cartilage 2015, 23, 933–939. 10.1016/j.joca.2015.02.011. 25689697

[ref46] Hatano R.; Fujii E.; Segawa H.; Mukaisho K.; Matsubara M.; Miyamoto K. I.; Hattori T.; Sugihara H.; Asano S. Ezrin, a Membrane Cytoskeletal Cross-Linker, Is Essential for the Regulation of Phosphate and Calcium Homeostasis. Kidney International 2013, 83 (1), 41–49. 10.1038/ki.2012.308. 22895514

[ref47] Merle B.; Garnero P. The Multiple Facets of Periostin in Bone Metabolism. Osteoporosis International 2012, 23, 1199–1212. 10.1007/s00198-011-1892-7. 22310955

[ref48] Bonnet N.; Garnero P.; Ferrari S. Periostin Action in Bone. Mol. Cell. Endocrinol. 2016, 432, 75–82. 10.1016/j.mce.2015.12.014. 26721738

[ref49] Schaffler M. B.; Choi K.; Milgrom C. Aging and Matrix Microdamage Accumulation in Human Compact Bone. Bone 1995, 17 (6), 521–525. 10.1016/8756-3282(95)00370-3. 8835305

[ref50] Burr D. B.; Turner C. H.; Naick P.; Forwood M. R.; Ambrosius W.; Sayeed Hasan M.; Pidaparti R. Does Microdamage Accumulation Affect the Mechanical Properties of Bone?. J. Biomech. 1998, 31 (4), 337–345. 10.1016/S0021-9290(98)00016-5. 9672087

[ref51] van Doorn N. L.; Wilson J.; Hollund H.; Soressi M.; Collins M. J. Site-Specific Deamidation of Glutamine: A New Marker of Bone Collagen Deterioration. Rapid Commun. Mass Spectrom. 2012, 26 (19), 2319–2327. 10.1002/rcm.6351. 22956324

[ref52] Cattaneo C.; Gelsthorpe K.; Phillips P.; Sokol R. J. Differential Survival of Albumin in Ancient Bone. Journal of Archaeological Science 1995, 22 (2), 271–276. 10.1006/jasc.1995.0029.

[ref53] Sawafuji R.; Cappellini E.; Nagaoka T.; Fotakis A. K.; Jersie-Christensen R. R.; Olsen J. v.; Hirata K.; Ueda S. Proteomic Profiling of Archaeological Human Bone. Royal Society Open Science 2017, 4 (6), 161004 10.1098/rsos.161004. 28680659 PMC5493901

[ref54] Rees S. G.; Hughes Wassell D. T.; Waddington R. J.; Embery G. Interaction of Bone Proteoglycans and Proteoglycan Components with Hydroxyapatite. Biochimica et Biophysica Acta (BBA) - General Subjects 2001, 1568 (2), 118–128. 10.1016/S0304-4165(01)00209-4. 11750759

[ref55] Nakamura M.; Takahashi I.; Echigo S.; Sasano Y. Versican and ADAMTSs Are Involved in Bone Development. International Congress Series 2005, 1284, 336–337. 10.1016/j.ics.2005.06.015.

[ref56] Coulson-Thomas Y. M.; Coulson-Thomas V. J.; Norton A. L.; Gesteira T. F.; Cavalheiro R. P.; Meneghetti M. C. Z.; Martins J. R.; Dixon R. A.; Nader H. B. The Identification of Proteoglycans and Glycosaminoglycans in Archaeological Human Bones and Teeth. PLoS One 2015, 10 (6), e0131105 10.1371/journal.pone.0131105. 26107959 PMC4481269

[ref57] Procopio N.; Hopkins R. J. A.; Harvey V. L.; Buckley M. Proteome Variation with Collagen Yield in Ancient Bone. J. Proteome Res. 2021, 20 (3), 1754–1769. 10.1021/acs.jproteome.0c01014. 33529527 PMC7944572

[ref58] Burr D. B. Changes in Bone Matrix Properties with Aging. Bone 2019, 120, 85–93. 10.1016/j.bone.2018.10.010. 30315999

[ref59] Pittner S.; Ehrenfellner B.; Monticelli F. C.; Zissler A.; Sänger A. M.; Stoiber W.; Steinbacher P. Postmortem Muscle Protein Degradation in Humans as a Tool for PMI Delimitation. International Journal of Legal Medicine 2016, 130 (6), 1547–1555. 10.1007/s00414-016-1349-9. 26951243 PMC5055573

[ref60] Raouf A.; Ganss B.; McMahon C.; Vary C.; Roughley P. J.; Seth A. Lumican Is a Major Proteoglycan Component of the Bone Matrix. Matrix Biology 2002, 21 (4), 361–367. 10.1016/S0945-053X(02)00027-6. 12128073

[ref61] Sztrolovics R.; Alini M.; Mort J. S.; Roughley P. J. Age-Related Changes in Fibromodulin and Lumican in Human Intervertebral Discs. Spine 1999, 24 (17), 1765 10.1097/00007632-199909010-00003. 10488504

[ref62] Robinson A. B.; McKerrow J. H.; Cary P. Controlled Deamidation of Peptides and Proteins: An Experimental Hazard and a Possible Biological Timer. Proc. Natl. Acad. Sci. U. S. A. 1970, 66 (3), 753–757. 10.1073/pnas.66.3.753. 5269237 PMC283114

[ref63] Robinson N. E.; Robinson A. B. Molecular Clocks. Proc. Natl. Acad. Sci. U. S. A. 2001, 98 (3), 944–949. 10.1073/pnas.98.3.944. 11158575 PMC14689

