# Supplementary material for: Insights into the Differential Preservation of Bone Proteomes in Inhumed and Entombed Cadavers from Italian Forensic Caseworks
Source: J Proteome Res. 2022 Mar 22;21(5):1285–98. doi: 10.1021/acs.jproteome.1c00904 (PMC9087355; doi:10.1021/acs.jproteome.1c00904)
Supplement: Supplementary file 1 — pr1c00904_si_001.pdf [file pr1c00904_si_001.pdf]

Supplementary Information

Insights into the Differential Preservation of Bone Proteomes in Inhumed and Entombed Cadavers from Italian Forensic Caseworks

Andrea Bonicelli[1], Aldo Di Nunzio[2], Ciro Di Nunzio[3], Noemi Procopio[1*]

[1]Forensic Science Research Group, Faculty of Health and Life Sciences, Applied Sciences, Northumbria University, NE1 8ST, Newcastle Upon Tyne, United Kingdom

[2]Chemical Sciences Department, University of Naples Federico II, 80126, Naples, Italy

[3]Legal Medicine Department, University of Catanzaro Magna Graecia, 88100, Germaneto, Italy

***Author for correspondence:** Noemi Procopio, noemi.procopio@northumbria.ac.uk, Ellison Building, Northumbria University, Newcastle Upon-Tyne, NE1 8ST, UK

Supplementary Data, Tables and Figures

Supplementary Table S1. Mean and standard deviations (SD) values for the proteins showing significant difference between the two burial conditions.

Supplementary Table S2. Correlation coefficients between each protein and both age and PMI.

Supplementary Figure S1. Error plots of proteins correlating significantly with PMI only.

Supplementary Figure S2. Error plots of proteins correlating significantly with age only.

Supplementary Data S1. Deamidation percentages for the six peptides highlighted in the study, in both zinc-lined (samples 1-8) and wooden (samples 9-14) coffins.

Supplementary Table S1. Mean and standard deviations (SD) values for the proteins showing significant difference between the two burial conditions. Significance: ***p<0.001 ***p*<0.01; **p*<0.05.

	**Zinc-lined coffin**			**Wooden coffin**			
	**N**	**Mean**	**SD**	**N**	**Mean**	**SD**	**Anova**
CLUS_HUMAN	16	13.96	0.471	12	13.35	0.572	F=9.529***
RCN3_HUMAN	16	12.69	0.807	12	11.92	1.279	F=3.805*
EMIL1_HUMAN	16	14.59	0.89	12	14.02	0.719	F=3.333*
GAS6_HUMAN	16	13.64	1.475	12	12.81	0.826	F=3.047*
LYOX_HUMAN	16	18.49	0.404	12	18.11	0.477	F=5.292**
B2MG_HUMAN	16	15.16	0.687	12	14.60	0.934	F=3.408*
SODE_HUMAN	16	16.38	0.874	12	15.85	0.705	F=2.961*
ANT3_HUMAN	16	14.13	1.068	12	13.26	1.417	F=3.411*
FLNC_HUMAN	16	13.35	0.876	12	10.92	0.722	F=61.211***
FINC_HUMAN	16	15.18	0.387	12	14.62	0.456	F=12.442***
APOA1_HUMAN	16	15.72	0.49	12	15.18	0.541	F=7.84***
MFGM_HUMAN	16	13.79	1.237	12	15.22	1.223	F=9.263***
A1AT_HUMAN	16	15.27	0.775	12	14.75	0.642	F=3.59*
POSTN_HUMAN	16	12.12	1.924	12	14.17	1.072	F=11.061***
TENA_HUMAN	16	14.59	0.401	12	14.16	0.371	F=8.181***
PPBT_HUMAN	16	16.12	0.326	12	15.82	0.391	F=5.12***
HTRA1_HUMAN	16	16.59	0.484	12	16.17	0.51	F=4.772***
NUCB2_HUMAN	16	16.38	0.598	12	15.82	0.309	F=8.529***
ENPL_HUMAN	16	15.47	0.437	12	14.92	0.449	F=10.463***
TETN_HUMAN	16	16.78	0.619	12	16.28	0.548	F=4.94**
OSTCN_HUMAN	16	23.3	0.55	12	22.79	0.654	F=4.913**
ZPI_HUMAN	16	15.71	0.626	12	15.22	0.653	F=4.055*
OLFL3_HUMAN	16	17.18	0.39	12	16.75	0.574	F=5.539**
TTHY_HUMAN	16	17.83	0.554	12	17.24	0.661	F=6.577**
COMA1_HUMAN	16	17.32	0.42	12	16.91	0.485	F=5.604**
COCA1_HUMAN	16	18.37	0.496	12	17.81	0.419	F=10.049***
LUM_HUMAN	16	22.45	0.541	12	22.00	0.575	F=4.486**
PGS1_HUMAN	16	22.79	0.506	12	22.36	0.557	F=4.485**

Supplementary Table S2. Correlation coefficients between each protein and both age and PMI. Significance: ****p<0.0001; ***p<0.001 **p<0.01; *p<0.05.

Spearman	PMI	Age
A1AT_HUMAN	-0.05	-0.05
A4_HUMAN	-0.34	-0.57**
ACTB_HUMAN	0.08	0.34
AEBP1_HUMAN	-0.12	0.16
ALBU_HUMAN	-0.50**	-0.59**
ANT3_HUMAN	0.39*	-0.01
APOA1_HUMAN	-0.19	-0.11
APOA4_HUMAN	-0.23	-0.31
ASPN_HUMAN	-0.56**	-0.41*
B2MG_HUMAN	-0.50**	-0.12
BMP1_HUMAN	-0.28	-0.15
CFAB_HUMAN	-0.1	-0.43*
CHAD_HUMAN	0.38*	-0.35
CLC11_HUMAN	-0.47*	-0.63***
CLUS_HUMAN	-0.2	-0.31
CO5A1_HUMAN	-0.23	-0.34
CO5A2_HUMAN	0.12	-0.28
CO9_HUMAN	-0.27	-0.57**
COBA2_HUMAN	-0.14	-0.46*
COCA1_HUMAN	0.24	0.61***
COMA1_HUMAN	-0.01	-0.41*
CSPG2_HUMAN	-0.68****	0.11
CTHR1_HUMAN	0.04	-0.31
DERM_HUMAN	0.22	0.25
ECM2_HUMAN	-0.04	-0.33
EMIL1_HUMAN	-0.05	-0.21
ENPL_HUMAN	0.1	0.09
EZRI_HUMAN	-0.08	-0.63***
FA10_HUMAN	-0.29	-0.05
FA7_HUMAN	0.33	-0.1
FA9_HUMAN	0.09	-0.03
FETUA_HUMAN	-0.44*	-0.39*
FINC_HUMAN	-0.23	-0.02
FLNC_HUMAN	-0.05	0.16
FMOD_HUMAN	-0.40*	-0.39*
G3P_HUMAN	-0.39*	0.03
GAS6_HUMAN	0.05	0.26
HBB_HUMAN	-0.13	0.12

HRG_HUMAN	-0.56**	-0.27
HTRA1_HUMAN	-0.36	-0.25
IBP5_HUMAN	-0.39*	-0.43*
IGG1_HUMAN	-0.24	-0.39*
IGKC_HUMAN	-0.25	-0.22
IGL1_HUMAN	-0.46*	-0.29
KAZD1_HUMAN	-0.31	-0.43*
KCRM_HUMAN	-0.05	-0.21
KNG1_HUMAN	-0.40*	-0.09
LUM_HUMAN	-0.12	-0.23
LYOX_HUMAN	-0.21	-0.11
MFGM_HUMAN	-0.28	-0.46*
MGP_HUMAN	-0.19	-0.13
MIME_HUMAN	-0.44*	-0.39*
MYH2_HUMAN	0.37	0.03
MYH7_HUMAN	0.16	0.01
NUCB1_HUMAN	-0.44*	-0.47*
NUCB2_HUMAN	-0.24	-0.06
OLFL3_HUMAN	0.12	0.03
OMD_HUMAN	-0.26	-0.62***
OSTCN_HUMAN	-0.23	-0.07
OSTP_HUMAN	-0.27	-0.36
PCOC1_HUMAN	-0.45*	-0.16
PDIA1_HUMAN	-0.32	-0.47*
PEDF_HUMAN	-0.21	-0.50**
PERM_HUMAN	-0.35	0.13
PGBM_HUMAN	-0.45*	-0.29
PGS1_HUMAN	-0.13	-0.26
PGS2_HUMAN	-0.14	0.04
POSTN_HUMAN	-0.32	-0.33
PPBT_HUMAN	-0.05	-0.2
PROC_HUMAN	-0.29	-0.69****
PROS_HUMAN	0.25	0.05
PROZ_HUMAN	0.1	0.3
RCN3_HUMAN	-0.40*	-0.14
SIAL_HUMAN	-0.01	0.04
SODE_HUMAN	-0.41*	0.29
SPP24_HUMAN	-0.32	0.35
SPRC_HUMAN	-0.37	-0.27
SRPX_HUMAN	-0.11	-0.11
TENA_HUMAN	0.17	-0.01
TETN_HUMAN	0.07	-0.31

TGFB1_HUMAN	-0.2	0.08
THRB_HUMAN	-0.02	-0.44*
TRH_HUMAN	-0.49**	-0.15
TSP1_HUMAN	-0.21	-0.25
TTHY_HUMAN	-0.47*	-0.29
VITRN_HUMAN	0.17	-0.37
VTNC_HUMAN	-0.06	0.28
ZPI_HUMAN	0.2	0.1

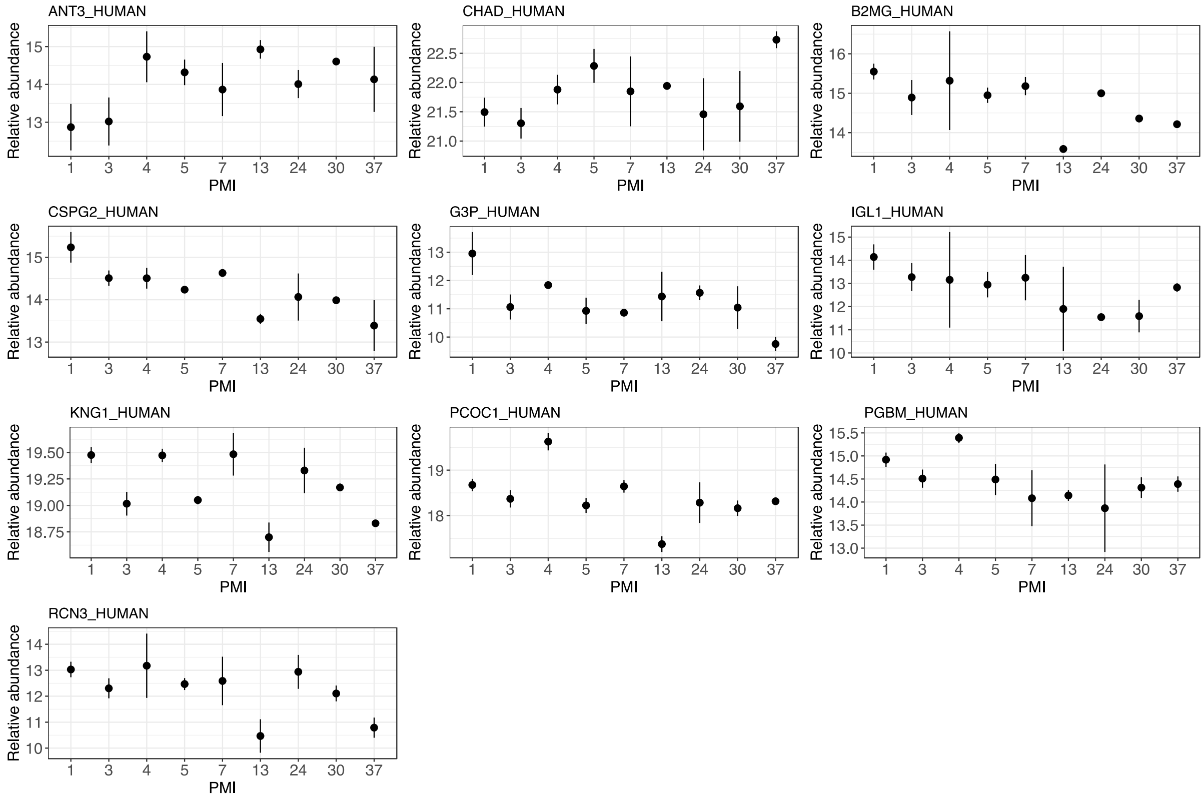

Supplementary Figure S1. Error plots of proteins correlating significantly with PMI only.

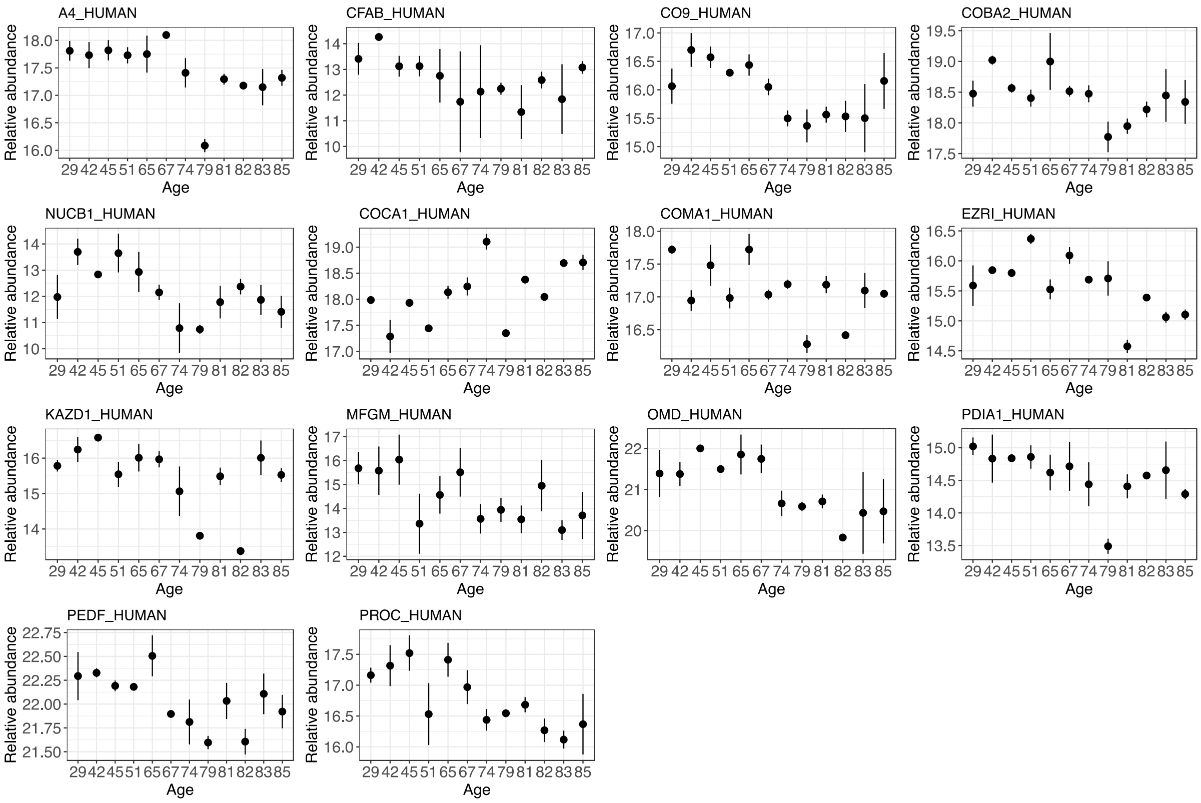

Supplementary Figure S2. Error plots of proteins correlating significantly with age only.
